# Perceived helpfulness of service sectors used for mental and substance use disorders: Findings from the WHO World Mental Health Surveys

**DOI:** 10.1186/s13033-022-00516-z

**Published:** 2022-01-29

**Authors:** Meredith G. Harris, Alan E. Kazdin, Richard J. Munthali, Daniel V. Vigo, Irving Hwang, Nancy A. Sampson, Ali Al-Hamzawi, Jordi Alonso, Laura Helena Andrade, Guilherme Borges, Brendan Bunting, Silvia Florescu, Oye Gureje, Elie G. Karam, Sing Lee, Fernando Navarro-Mateu, Daisuke Nishi, Charlene Rapsey, Kate M. Scott, Juan Carlos Stagnaro, Maria Carmen Viana, Bogdan Wojtyniak, Miguel Xavier, Ronald C. Kessler

**Affiliations:** 1 School of Public Health, The University of Queensland, Level 2, Public Health Building (887), 288 Herston Road, Herston, QLD 4006 Australia; 2 Queensland Centre for Mental Health Research, The Park Centre for Mental Health, Wolston Park Rd, Wacol, QLD 4076 Australia; 3 Department of Psychology, Yale University, 2 Hillhouse Avenue- 208205, New Haven, CT 06520 USA; 4 Department of Psychiatry, University of British Columbia, UBC Hospital—Detwiller Pavilion, UBC Vancouver Campus, Room 2813, 2255 Wesbrook Mall, Vancouver, BC V6T 2A1 Canada; 5 Department of Global Health and Social Medicine, Harvard Medical School, 641 Huntington Avenue, Boston, MA 02115 USA; 6 Department of Health Care Policy, Harvard Medical School, 180 Longwood Avenue, Boston, MA 02115 USA; 7 College of Medicine, Al-Qadisiya University, Al-Diwaniyah, P.O.Box 88, Al-Qadisiyah, Iraq; 8 IMIM-Hospital del Mar Medical Research Institute, PRBB Building, Doctor Aiguader, 88, 08003 Barcelona, Spain; 9 CIBER en Epidemiología Y Salud Pública (CIBERESP), Av. Monforte de Lemos, 3-5, Pabellón 11, Planta 0, 28029 Madrid, Spain; 10 Pompeu Fabra University (UPF), Plaça de la Mercè, 10-12, 08002 Barcelona, Spain; 11 University of São Paulo Medical School, Núcleo de Epidemiologia Psiquiátrica - LIM 23, Rua Dr. Ovidio Pires de Campos, 785, São Paulo, CEP 05403-010 Brazil; 12 National Institute of Psychiatry Ramón de La Fuente Muñiz, Calzada México-Xochimilco, 101, Colonia San Lorenzo Huipulco, DF 14370 México City, Mexico; 13 School of Psychology, Ulster University, College Avenue, Londonderry, BT48 7JL UK; 14 National School of Public Health, Management and Development, 31 Vaselor Str, 21253 Bucharest, Romania; 15 Department of Psychiatry, University of Ibadan, University College Hospital, Ibadan, 5116 PMB Nigeria; 16 Institute for Development, Research, Advocacy and Applied Care (IDRAAC), Achrafieh, St. George Hospital Street, Beirut, Lebanon; 17 Department of Psychiatry and Clinical Psychology, St George Hospital University Medical Center, Ashrafieh, Beirut, 166378 Lebanon; 18 Faculty of Medicine, Balamand University, Rond Point Saloumeh, Sin el Fil, Beirut, Lebanon; 19 Department of Psychiatry, Chinese University of Hong Kong, Tai Po, Hong Kong; 20 G/F Multicentre, Tai Po Hospital, 9 Chuen On Road, Tai Po, Hong Kong; 21 Unidad de Docencia, Investigacion Y Formación en Salud Mental, Servicio Murciano de Salud, Murcia Health Service, C/ Lorca, nº 58. -El Palmar, 30120 Murcia, Spain; 22 Instituto Murciano de Investigación Biosanitaria Virgen de La Arrixaca, El Palmar, 30120 Murcia, Spain; 23 Centro de Investigación Biomédica en ERed en Epidemíologia Y Salud Pública, El Palmar, 30120 Murcia, Spain; 24 Department of Mental Health, Graduate School of Medicine, The University of Tokyo, 7-3-1, Hongo, Bunkyo-ku, Tokyo, 113-0033 Japan; 25 Department of Psychological Medicine, University of Otago, PO Box 56, Dunedin, 9054 New Zealand; 26 Departamento de Psiquiatría Y Salud Mental, Facultad de Medicina, Universidad de Buenos Aires, 2155, C1121ABG CABA Paraguay, Argentina; 27 Department of Social Medicine, Postgraduate Program in Public Health, Federal University of Espírito Santo, Rua Dr. Euríco de Águiar, 888/705, Vitoria, Espirito Santo—ES 29052-600 Brazil; 28 National Institute of Public Health, National Research Institute, 24 Chocimska St., 00-791 Warsaw, Poland; 29 Lisbon Institute of Global Mental Health and Chronic Diseases Research Center (CEDOC), Universidade Nova de Lisboa, Campo dos Mártires da Pátria, 130, 1169-056 Lisbon, Portugal

**Keywords:** Mental health services, Health service use, Perceived helpfulness, Patient perspectives, Healthcare providers, Service sectors, Treatment profiles, Mental disorders, Substance use disorders

## Abstract

**Background:**

Mental healthcare is delivered across service sectors that differ in level of specialization and intervention modalities typically offered. Little is known about the perceived helpfulness of the combinations of service sectors that patients use.

**Methods:**

Respondents 18 + years with 12-month DSM-IV mental or substance use disorders who saw a provider for mental health problems in the year before interview were identified from WHO World Mental Health surveys in 17 countries. Based upon the types of providers seen, patients were grouped into nine mutually exclusive single-sector or multi-sector ‘treatment profiles’. Perceived helpfulness was defined as the patient’s maximum rating of being helped (‘a lot’, ‘some’, ‘a little’ or ‘not at all’) of any type of provider seen in the profile. Logistic regression analysis was used to examine the joint associations of sociodemographics, disorder types, and treatment profiles with being helped ‘a lot’.

**Results:**

Across all surveys combined, 29.4% (S.E. 0.6) of respondents with a 12-month disorder saw a provider in the past year (N = 3221). Of these patients, 58.2% (S.E. 1.0) reported being helped ‘a lot’. Odds of being helped ‘a lot’ were significantly higher (odds ratios [ORs] = 1.50–1.89) among the 12.9% of patients who used specialized multi-sector profiles involving both psychiatrists and other mental health specialists, compared to other patients, despite their high comorbidities. Lower odds of being helped ‘a lot’ were found among patients who were seen only in the general medical, psychiatrist, or other mental health specialty sectors (ORs = 0.46–0.71). Female gender and older age were associated with increased odds of being helped ‘a lot’. In models stratified by country income group, having 3 or more disorders (high-income countries only) and state-funded health insurance (low/middle-income countries only) were associated with increased odds of being helped ‘a lot’.

**Conclusions:**

Patients who received specialized, multi-sector care were more likely than other patients to report being helped ‘a lot’. This result is consistent with previous research suggesting that persistence in help-seeking is associated with receiving helpful treatment. Given the nonrandom sorting of patients by types of providers seen and persistence in help-seeking, we cannot discount that selection bias may play some role in this pattern.

**Supplementary Information:**

The online version contains supplementary material available at 10.1186/s13033-022-00516-z.

## Introduction

Hundreds of millions of people experience mental and substance use disorders worldwide each year [1, 2]. Across countries where treatment rates have been measured, up to one-third of adults with these disorders see a provider for treatment in a year [3]. A plethora of treatment studies have shown various interventions and service models to be efficacious or effective for these disorders [4–7]. Although a great many evidence-based treatments have been documented to be associated with aggregate reductions in symptoms, these evaluations seldom ask patients whether they perceived their treatment to be helpful [6, 8, 9]. This is a meaningful omission because existing studies focus largely on outcomes that clinicians consider important in evaluating treatments, whereas the perceptions of patients might be different. The patient’s perspective is increasingly seen as an important independent perspective on treatment [10, 11].

In particular, the patient’s perception of treatment helpfulness is now recognized as a meaningful indicator of healthcare quality in its own right [12] and has been associated with desirable treatment process indicators including more frequent health care use [13], retention in treatment [14, 15] and longer duration of treatment [16]. Measures of perceived helpfulness can complement measures of symptom response and quality of life that are typically used in treatment trials, but which may not capture changes in functioning or other specific problems that prompted the patient to seek treatment in the first place [6, 17]. Moreover, routine reporting of patient perceptions such as perceived helpfulness may provide a credible source of information for potential help-seekers wanting to understand what to expect from treatment [8] and improve the public accountability of health services [18].

To date, perceived helpfulness has been evaluated primarily in small-scale studies of a single intervention or from one or a few clinics, services, or mental health professionals [9, 19]. Randomized controlled trials of treatment do well in isolating the impact of specific interventions. However, they do not reflect, nor are they intended to reflect, how patients negotiate and receive services. This is important because, in the real world, mental health care is provided by a wide range of providers. These providers represent different service sectors that vary in their level of specialization and capacity to deliver the kinds of interventions appropriate for different types of disorders and levels of need [20, 21]. Moreover, these service sectors share patient care to greater or lesser extent [22]. Comparing perceived helpfulness across the specific combinations of service sectors that people actually use could help to identify systematic disparities in the quality of mental healthcare and inform ways to better triage and personalize treatment.

Epidemiologic surveys are well-suited to evaluating variations in perceived helpfulness in broadly defined populations but, to date, perceived helpfulness has been a relatively understudied topic [23]. In available studies, approximately half to two-thirds of patients said that the provider(s) they saw for mental health or substance use problems in the past year helped them ‘a lot’ or ‘extremely’ [24, 25]. A few studies have examined whether ratings of perceived helpfulness differ between patients seen in the general medical sector and those seen in the specialized mental health sector, the latter group usually combining psychiatrists with non-medical mental health professionals. Some analyses have shown similar levels of perceived helpfulness across these sectors, but without taking account the complexity of patients’ problems or possible overlap in providers [13, 14]. In contrast, other analyses have shown treatment in the specialized mental health sector to be perceived as more helpful than treatment only in the general medical sector, after controlling for severity and comorbidity [24, 26]. Although these findings are informative, they are based either on individual provider types or combinations of provider types that differ in the treatment modalities they can offer. Moreover, these two service sectors do not reflect the full spectrum of service sectors that people use; for example, many people consult human services providers, spiritual providers and healers [22, 27–29]. A study of the perceived helpfulness of more nuanced combinations of service sectors, hereafter referred to as treatment profiles, is needed [18].

In this study, we explored variations in perceived helpfulness of the treatment profiles used by patients with 12-month mental and substance use disorders across 17 countries. Our aims were to: (1) describe the array of treatment profiles through which mental health care is delivered; (2) examine variations in perceived helpfulness across treatment profiles; (3) identify patient-level social and clinical characteristics associated with using each of these treatment profiles; and (4) examine the extent to which patient-level characteristics and treatment profiles are associated with perceived helpfulness.

## Methods

### Samples and procedures

The World Health Organization (WHO) World Mental Health (WMH) surveys are a coordinated set of epidemiological surveys that provide cross-national data on the prevalence, correlates and treatment of mental and substance use disorders [30, 31]. This reported uses data from WMH surveys in 17 countries, including 9 classified as high-income countries at the time of data collection (Argentina, New Zealand, Northern Ireland, Poland, Portugal, Saudi Arabia, Spain—Murcia, Japan, and United States) and 8 classified as low- or middle-income countries (Brazil—São Paulo, Bulgaria, Colombia—Medellin, Iraq, Mexico, People’s Republic of China—Shenzen, Peru, and Romania). Nine surveys were nationally representative, and the remainder were representative of selected regions, metropolitan areas or urbanised areas (Table 1).

**Table 1:** World Mental Health sample characteristics by World Bank income categories^a^

Country by income category	Survey^b^	Sample characteristics^c^	Field Dates	Age range	Sample Size Part II	Response rate (%)^d^	Any DSM-IV 12-month disorder among the Part II sample		12-month use of providers for mental health among those with any DSM-IV 12-month disorder	
							%	SE	%	SE
I. Low/middle-income countries										
Brazil—São Paulo	São Paulo Megacity	São Paulo metropolitan area	2005–8	18–93	2942	81.3	21.5	0.7	22.8	1.3
Bulgaria 2	NSHS—2	Nationally representative	2016–17	18–91	578	61.0	6.1	1.4	47.7	6.7
Colombia—Medellin	MMHHS	Medellin metropolitan area	2011–12	19–65	1673	97.2	15.2	1.2	15.8	2.0
Iraq	IMHS	Nationally representative	2006–7	18–96	4332	95.2	8.1	0.6	1.2	0.5
Mexico	M-NCS	All urban areas of the country (approximately 75% of the total national population)	2001–2	18–65	2362	76.6	11.0	0.8	17.1	1.9
Peru	EMSMP	Five urban areas of the country (approximately 38% of the total national population)	2004–5	18–65	1801	90.2	9.5	0.6	17.2	2.2
PRC—Shenzhen^e^	Shenzhen	Shenzhen metropolitan area. Included temporary residents as well as household residents	2005–7	18–88	2475	80.0	3.8	0.5	4.2	0.7
Romania	RMHS	Nationally representative	2005–6	18–96	2357	70.9	5.7	0.5	20.4	2.6
Total					18,520	81.9	10.4	0.3	16.8	0.7
II. High-income countries										
Argentina	AMHES	Eight largest urban areas of the country (approximately 50% of the total national population)	2015	18–98	2116	77.3	11.5	0.7	26.4	2.8
Japan	WMHJ 2002–2006	Eleven metropolitan areas	2002–6	20–98	1682	55.1	6.1	0.6	26.0	2.6
New Zealand^e^	NZMHS	Nationally representative	2004–5	18–98	7312	73.3	18.8	0.5	34.3	1.3
Northern Ireland	NISHS	Nationally representative	2005–8	18–97	1986	68.4	22.5	1.3	49.7	2.5
Poland	EZOP	Nationally representative	2010–11	18–65	4000	50.4	8.5	0.4	17.9	1.7
Portugal	NMHS	Nationally representative	2008–9	18–81	2060	57.3	19.3	1.0	35.9	2.3
Saudi Arabia^e^	SNMHS	Nationally representative	2013–16	18–65	1793	61.0	11.4	1.1	15.6	2.8
Spain-Murcia	PEGASUS- Murcia	Murcia region. Regionally representative	2010–12	18–96	1459	67.4	12.7	0.9	40.9	3.0
United States	NCS-R	Nationally representative	2001–3	18–99	5692	70.9	22.3	0.8	38.3	1.1
Total					28,100	63.5	14.7	0.3	34.7	0.8
III. Pooled across all countries					46,620	69.4	13.9	0.2	29.4	0.6
Between countries, *X*^2^_16_ (p-value)							1542.34 (< 0.001)*		3177.29 (< 0.001)*	
Low/middle-income countries vs. high-income countries, *X*^2^_1_ (p-value)							1081.29 (< 0.001)*		837.55 (< 0.001)*	

*DSM-IV* Diagnostic and Statistical Manual of Mental Disorders 4th edition, *PRC* People’s Republic of China

^*^Significant at .05 level, two-sided test

^a^The World Bank (2012) Data. Accessed May 12, 2012 at: https://data.worldbank.org/country. Some of the WMH countries have moved into new income categories since the surveys were conducted. The income groupings above reflect the status of each country at the time of data collection. The current income category of each country is available at the preceding URL

^b^NSHS (Bulgaria National Survey of Health and Stress); MMHHS (Medellín Mental Health Household Study); IMHS (Iraq Mental Health Survey); M-NCS (The Mexico National Comorbidity Survey); EMSMP (La Encuesta Mundial de Salud Mental en el Peru); RMHS (Romania Mental Health Survey); AMHES (Argentina Mental Health Epidemiologic Survey); WMHJ 2002–2006 (World Mental Health Japan Survey); NZMHS (New Zealand Mental Health Survey); NISHS (Northern Ireland Study of Health and Stress); EZOP (Epidemiology of Mental Disorders and Access to Care Survey); NMHS (Portugal National Mental Health Survey); SNMHS (Saudi National Mental Health Survey); PEGASUS-Murcia (Psychiatric Enquiry to General Population in Southeast Spain-Murcia); NCS-R (The US National Comorbidity Survey Replication)

^c^Most WMH surveys are based on stratified multistage clustered area probability household samples in which samples of areas equivalent to counties or municipalities in the US were selected in the first stage followed by one or more subsequent stages of geographic sampling (e.g., towns within counties, blocks within towns, households within blocks) to arrive at a sample of households, in each of which a listing of household members was created and one or two people were selected from this listing to be interviewed. No substitution was allowed when the originally sampled household resident could not be interviewed. These household samples were selected from Census area data. In Poland and Spain-Murcia, respondents were selected from municipal, country resident or universal health-care registries, without listing households. The Japanese sample is the only totally un-clustered sample, with households randomly selected in each of the 11 metropolitan areas and one random respondent selected in each sample household. 9 of the 17 surveys are based on nationally representative household samples

^d^The response rate is calculated as the ratio of the number of households in which an interview was completed to the number of households originally sampled, excluding from the denominator households known not to be eligible either because of being vacant at the time of initial contact or because the residents were unable to speak the designated languages of the survey. The weighted average response rate is 69.4%

^e^For the purposes of cross-national comparisons we limit the sample to those 18 +

Interviews were administered face-to-face by trained, lay interviewers in respondents’ homes. The interview schedule and training materials were developed in English and translated into other languages using a standardised translation protocol [32]. Interviewers completed a certification course before commencing fieldwork and standardised quality control tools were applied to monitor interviewer accuracy [33]. Informed consent was obtained prior to beginning the interview. Procedures for obtaining informed consent and protecting respondents were approved and monitored by Institutional Review Boards of the organizations coordinating the surveys in each country.

Interviews were administered in two parts to reduce respondent burden. Part I assessed core Diagnostic and Statistical Manual of Mental Disorders, Fourth Edition (DSM-IV) disorders and was administered to all respondents. Part II assessed additional disorders and correlates and was administered to all respondents who met lifetime criteria for any Part I disorder and to a probability subsample of other Part I respondents. Part II data were weighted to adjust for differential probabilities of selection into Part II and deviations between the sample population demographic-geographic distributions [34].

### Measures

#### Diagnoses

Twelve-month diagnoses were generated according to DSM-IV criteria using the WHO's Composite International Diagnostic Interview Version 3.0 (CIDI 3.0) [23], a fully structured lay‐administered diagnostic interview. Diagnoses included in this report were mood disorders (major depressive disorder, bipolar disorder), anxiety disorders (panic disorder/agoraphobia, generalized anxiety disorder, social phobia, specific phobia, posttraumatic stress disorder), and substance use disorders (alcohol and illicit drug abuse with or without dependence). DSM-IV organic exclusion rules were applied. Clinical reappraisal studies have shown generally good concordance between diagnoses based on the CIDI 3.0 and blinded clinical reappraisal interviews [35, 36].

#### Service sectors and treatment profiles

All Part II respondents were asked if they had ever seen any type of provider for problems with emotions, nerves, mental health, or use of alcohol or drugs. If so, they were asked whether, in the 12 months before interview, they had seen providers in the following five service sectors: General medical, including a general practitioner/primary care doctor or other medical doctor; Psychiatrist; Other mental health specialty, including a psychologist, any other mental health professional in any setting, a social worker or counselor in a mental health specialized setting; Other health provider, including a social worker or counselor in a human services setting, or another non-medical health professional; and Spiritual/healer, including a spiritual advisor or healer. To aid recall, examples of these types of providers were presented in a respondent booklet; these examples varied somewhat across countries to reflect local circumstances. Use of other services such as self-help groups, internet self-help applications and hotlines was not included in the current report because questions about the helpfulness of these services were not asked in the survey.

Among respondents who had used these service sectors in the past year, we then defined their use of 9 mutually exclusive single-sector and multi-sector 12-month ‘treatment profiles’. We started by calculating the probabilities of use of all possible combinations of sectors. We found that 91.8% (weighted) of respondents were seen in 9 treatment profiles. The remaining 8.2% (weighted) were seen in rare combinations (ranging from < 0.1% to 1.6%) that always involved the Other health provider and/or Spiritual/healer sectors. These rare combinations were recoded into the 9 mutually exclusive profiles (see Additional file 1: Table A1 for details of how rare combinations were recoded). The 9 treatment profiles broadly reflect the level of mental health specialization and key intervention modalities (pharmacotherapies and psychotherapies) that may be offered by the included providers. Specifically, the General medical with Psychiatrist, General medical with Other mental health specialty, General medical with Psychiatrist and Other mental health specialty, Psychiatrist-only and Psychiatrist with Other mental health speciality profiles could potentially deliver combined pharmacotherapies and psychotherapies; the General medical-only and General medical with Spiritual/healer profiles are more likely to involve pharmacotherapies; the Other mental health specialty-only profile could deliver psychotherapies; and the Spiritual/healer-only profile is likely to involve neither pharmacotherapy or psychotherapy [22].

#### Perceived helpfulness

Respondents who had seen a professional in the 12 months before interview were asked ‘Did [the professional] help you a lot, some, a little, or not at all?’ We dichotomised ratings of perceived helpfulness (‘a lot’ versus ‘some’, ‘a little’ or ‘not at all’) as we considered being helped ‘a lot’ as most congruent with patient-centered care [12]. If more than one type of provider was seen, we applied the maximum rating of helpfulness for any provider seen. This means that our measure of perceived helpfulness represents the cumulative probability of being helped ‘a lot’, even if that required contact with multiple providers. We considered this an appropriate approach because we were interested in the maximum results patients obtained from their contact with the mental health system. Some other studies have explored average helpfulness across providers or the helpfulness of the most frequently seen provider, however these approaches would potentially underestimate the probability of being helped ‘a lot’ by *any* provider seen [14, 25].

#### Predictors

Sociodemographic predictors were gender, age at interview (≤ 34, 35–49, 50–64, ≥ 65 years), marital status (married/cohabiting, separated/widowed/divorced, never married), employment (working, student, homemaker, retired, other), type of health insurance (state-funded or subsidized, insurance through an employer or national social security, direct private/optional insurance, any other health insurance, no insurance coverage or unknown), family income and education (each coded low, low-average, high-average, high). To account for wide cross-national variations in family income and education, country-specific coding schema were used. In high-income countries, the high education category corresponded to a college degree, high-average to some post-secondary education without a college degree, low-average to secondary school graduation, and low to less than secondary education. These four categories comprised roughly equal sized groups. Thresholds in other countries were applied to achieve the same split. For family income, we classified high income as greater than two times the within-country median per capita family income (i.e. income divided by number of family members), high-average income as 100–200% times the median, low-average as 50–100% of the median, and low income as less than 50% of the median. Clinical predictors were each of the eight 12-month diagnoses and a variable representing number of diagnoses (exactly 1, exactly 2, 3 or more). This allowed us to capture type and amount of mental or substance use disorder comorbidity, which is important because comorbidity may complicate diagnosis, complicate treatment, and intensify functional impairment [37], any of which may influence the outcome of treatment. Treatment-related predictors were the 9 treatment profiles.

### Analysis methods

Cross-tabulations were used to examine treatment distributions and their associations with sociodemographics and disorder types as well as with the distributions of perceived helpfulness across treatment profiles. Logistic regression analysis was then used to examine the joint associations of sociodemographics, disorder types, and treatment profiles with a dichotomous patient report of being helped ‘a lot’. Logits were exponentiated and are reported as odds-ratios (ORs) with their 95% confidence intervals. The ORs associated with treatment profiles were centered to have a product of 0, allowing direct interpretation of each individual OR with the average in the total sample. That is, the odds of being helped ‘a lot’ for each treatment profile could then be compared to the weighted average of being helped a ‘lot’ for all treatment profiles combined. Interactions were estimated between sociodemographics and disorder types, sociodemographics and treatment profiles, and between disorder types and treatment profiles to determine whether joint associations were additive. Analyses were also replicated separately in high-income countries and low/middle-income countries. Statistical significance was consistently evaluated using 0.05 level two-sided design-based tests.

## Results

### Sample characteristics

Survey characteristics are shown in Table 1. The weighted average response rate across all surveys was 69.4%. The total sample comprised 46,620 respondents aged 18 years and over. Across all surveys combined, 13.9% of respondents met criteria for any of the 12-month disorders included in this study. This report focuses on the 29.4% (N = 3,221) of respondents with a 12-month disorder who had seen a provider in the year before interview. The probability of seeing a provider was, on average, 2.1 times higher in high-income than in low/middle-income surveys (34.7% vs. 16.8%, χ^2^_1_ = 837.55, p < 0.001).

### Service sectors and treatment profiles

Table 2 shows the distribution of contact with providers grouped into service sectors and treatment profiles. Keeping in mind that patients may have had contact with more than one service sector, the majority had contact with the General medical sector (60.9%). Fewer had contact with the Other mental health specialty (37.0%) or Psychiatrist (29.7%) sectors and fewer yet with the Spiritual/healer sector (17.5%). Only a small percentage (2.2%) had contact with the Other health provider sector.

**Table 2:** Distributions of treatment and distributions of perceived helpfulness across service sectors and treatment profiles, among respondents with 12-month DSM-IV disorders who reported 12-month use of providers for mental health (N = 3221)

	Distribution of treatment			Perceived helpfulness (maximum)^a^										
				‘A lot’		‘Some’		‘A little’		‘Not at all’		Test for Equal Proportions		
	n	%	SE	%	SE	%	SE	%	SE	%	SE	*X*^2^	df	p-value
I. Service sectors^b^														
General medical	1920	60.9	0.9	58.1	1.3	24.7	1.2	10.8	0.1	6.5	0.7	765.98*	3	< 0.001
Psychiatrist	968	29.7	0.9	62.6	1.6	22.8	1.5	9.0	1.1	5.7	0.9	466.30*	3	< 0.001
Other mental health speciality	1187	37.0	1.0	64.7	1.6	22.5	1.4	8.3	1.0	4.6	0.6	646.17*	3	< 0.001
Other health provider	72	2.2	0.3	70.2	5.7	22.1	5.3	5.5	1.3	2.4	2.4	23.89*	3	< 0.001
Spiritual/healer	566	17.5	0.9	77.3	2.3	16.6	2.1	4.6	0.9	1.5	0.5	553.61*	3	< 0.001
II. Treatment profiles^c^														
General medical-only	1111	35.2	1.1	48.8	1.7	27.3	1.5	14.7	1.4	9.3	1.1	249.46*	3	< 0.001
Psychiatrist-only	386	11.6	0.7	51.3	3.0	24.6	2.5	13.7	1.9	10.4	2.0	91.84*	3	< 0.001
Other mental health specialty-only	458	13.8	0.7	55.5	2.5	24.3	2.2	11.4	1.6	8.9	1.5	145.42*	3	< 0.001
Spiritual/healer-only	265	7.8	0.6	66.7	3.6	22.6	3.5	7.9	1.6	2.8	0.9	150.98*	3	< 0.001
General medical with Psychiatrist	170	5.1	0.4	64.1	4.3	24.6	3.8	6.7	1.8	4.6	1.8	89.08*	3	< 0.001
General medical with Other mental health specialty	317	10.3	0.6	67.6	3.0	22.5	2.9	7.6	1.8	2.3	0.8	192.13*	3	< 0.001
General medical with Spiritual/healer	102	3.3	0.4	82.7	4.2	12.6	3.0	3.4	3.0	1.3	1.3	112.91*	3	< 0.001
Psychiatrist with Other mental health specialty	192	5.9	0.5	68.6	4.0	20.3	3.3	9.4	2.8	1.7	0.4	123.55*	3	< 0.001
General medical with Psychiatrist and Other mental health specialty	220	7.0	0.6	75.0	2.9	20.6	2.6	2.5	1.1	1.9	1.0	192.13*	3	< 0.001
III. Total (all profiles combined)	3221	100.0	-	58.2	1.0	24.2	0.9	10.9	0.7	6.7	0.5	1257.42*	3	< 0.001
Perceived helpfulness (4 categories) across all profiles												102.86*	24	< 0.001
Helped 'a lot' vs. 'some'/'a little'/'not at all' across all profiles												78.97*	8	< 0.001
Helped 'a lot'/'some' vs. 'a little'/'not at all' across all profiles												68.90*	8	< 0.001
Helped 'a lot'/'some'/'a little' vs. 'not at all' across all profiles												36.98*	8	< 0.001

^*^Significant at .05 level, two-sided test

^a^The maximum rating of how much the patient said they were helped by any type of provider seen

^b^Service sectors: General medical (general practitioner/primary care doctor or other medical doctor); Psychiatrist; Other mental health specialty (psychologist, any other mental health professional in any setting, a social worker or counselor in a mental health specialized setting); Other health provider (social worker or counselor in a human services setting, or a non-medical health professional); and Spiritual/healer (spiritual advisor or healer)

^c^Treatment profiles represent the 9 most commonly used combinations of service sectors. These 9 profiles accounted for 91.8% of respondents; rare combinations of indvidual sectors were recoded into one of the 9 profiles (see Additional file 1: Table A1)

With respect to the more granular, mutually exclusive treatment profiles, the 3 most commonly used profiles were the single-sector General medical-only (35.2%), Other mental health specialty-only (13.8%) and Psychiatrist-only (11.6%) profiles. The remaining profiles were each used by 3.3%-10.3% of respondents. Notably, 12.9% of patients used a specialized multi-sector profile involving psychiatrists and other mental health specialists (5.9% without the General medical sector and 7.0% with the General medical sector).

### Helpfulness of service sectors and treatment profiles

Table 2 also shows the distribution of ratings of helpfulness. Across all treatment profiles combined, 58.2% of patients said they were helped ‘a lot’ by the professionals they saw, 24.2% ‘some’, 10.9% ‘a little’ and 6.7% ‘not at all’. This pattern of decreasing proportions from greatest to least helpfulness was found within each profile, even though the exact proportions in each category varied across profiles (χ^2^_24_ = 102.86, p < 0.001). Differences across profiles also existed when response categories were collapsed in various ways. Notably, the proportion of patients reporting being helped ‘a lot’ differed across profiles (χ^2^_8_ = 78.97, p < 0.001), and was lower in the three most commonly used profiles (General medical-only, Psychiatrist-only, Other mental health specialty-only; 48.8%-55.5%) than the other profiles (64.1%-82.7%). The distribution of helpfulness ratings did not vary significantly according to number of 12-month disorders (χ^2^_6_ = 8.04, p = 0.240) (Additional file 1: Table A2).

Further, Table 2 shows the incremental effect of using each additional type of sector, over and above using a single sector only. For example, 48.8% of patients who used the General-medical only profile said they were helped ‘a lot’. The percentage was higher among those who used the 2-sector General medical with Psychiatrist (64.1%), and General medical with Other mental health specialty (67.6%) profiles, and the 3-sector General medical sector with both Psychiatrist and Other mental health specialty profile (75.0%). However, the highest percentage was among those who used the 2-sector General medical with Spiritual/healer (82.7%) profile. These patterns indicate that significant numbers of people said the additional sector(s) helped ‘a lot’ over and above the first sector, but that the exact percentage depended on the specific combination of sectors used, not just the number of sectors used.

### Correlates of treatment profiles

All patient sociodemographic characteristics differed significantly across treatment profiles, with the exception of education (Additional file 1: Table A3). Mental disorder prevalence also differed significantly across treatment profiles. Both sets of patterns are complex, but one especially noteworthy pattern was that patients with more complex comorbidities were more likely than others to have used the multi-sector General medical with Psychiatrist and Other mental health specialty profile (χ^2^_2_ = 42.49, p < 0.001), the General medical with Psychiatrist profile (χ^2^_2_ = 11.40, p = 0.003) and the General medical with Other mental health specialty profile (χ^2^_2_ = 13.51, p < 0.001), whereas patients with single disorders were more likely than others to have used the single-sector General medical only (χ^2^_2_ = 19.81, p < 0.001) and the Other mental health specialty-only (χ^2^_2_ = 20.92, p < 0.001) profiles.

### Predictors of being helped ‘a lot’

The logistic regression model predicting the perception of being helped ‘a lot’ found that gender (χ^2^_1_ = 5.40, p = 0.020) and age at interview (χ^2^_2_ = 17.34, p = 0.001) were the only significant sociodemographic correlates, with males significantly less likely than females to report that they were helped ‘a lot’ and a nonmonotonic association of age with being helped ‘a lot’ (older patients aged 65 years and over more likely than patients younger than 50 years) (Table 3). None of the mental disorders considered was a significant correlate of being helped ‘a lot’ (χ^2^_1_ = 0.04–3.66, p = 0.845–0.056). However, the disorder variables were significant as a set (χ^2^_8_ = 18.53, p = 0.018). It is important to remember, in interpreting this pattern, that all patients had at least one disorder and could have multiple disorders. This means that individual ORs represent incremental associations of each disorder with perceived helpfulness. These ORs were for the most part negative, which means that comorbidities were for the most part associated with reduced relative-odds of perceived helpfulness, although none of these was individually significant. We also initially included a term for 3 or more disorders, but it was not significant and therefore not included in the final model.

**Table 3:** Logistic regression results showing joint associations of sociodemographics, disorder types, and treatment profiles with perceived helpfulness (being helped 'a lot'), among respondents with 12-month DSM-IV disorders who reported 12-month use of providers for mental health (N = 3119)^a^

	Perceived helpfulness (being helped 'a lot')^b^					
	OR	95% CI		*X*^2^	df	p-value
Gender (ref: Female)	Reference			5.40*	1	0.020
Male	0.76	0.60	0.96			
Age at interview (years) (ref: ≥ 65)	Reference			17.34*	3	0.001
≤ 34 years	0.61	0.36	1.02			
35–49	0.77	0.49	1.21			
50–64	1.13	0.74	1.73			
Marital status (ref: Married/cohabitating)	Reference			0.54	2	0.764
Separated/widowed/divorced	0.94	0.76	1.17			
Never married	1.04	0.81	1.34			
Family income^c^ (ref: High)	Reference			1.54	3	0.672
Low	0.87	0.66	1.14			
Low-average	0.89	0.70	1.13			
High-average	0.87	0.67	1.13			
Education^d^ (ref: High)	Reference			1.55	3	0.671
Low	1.04	0.77	1.40			
Low-average	0.98	0.75	1.29			
High-average	0.89	0.70	1.12			
Employment (ref: Working)	Reference			6.70	4	0.153
Homemaker	0.85	0.63	1.15			
Retired	1.07	0.72	1.60			
Student	0.79	0.50	1.24			
Other	0.73	0.56	0.95			
Insurance (ref: None or unknown)	Reference			6.38	4	0.172
State funded coverage or subsidized insurance	1.33	0.93	1.92			
Insurance through employment or national social security	1.49	0.98	2.27			
Direct private/optional insurance	0.85	0.44	1.67			
Other	1.32	0.90	1.93			
12-month DSM-IV disorders						
Major depressive disorder (ref: No)	0.82	0.67	1.01	3.66	1	0.056
Bipolar disorder (ref: No)	0.73	0.52	1.02	3.41	1	0.065
Generalized anxiety disorder (ref: No)	0.82	0.63	1.08	2.00	1	0.157
Panic disorder/Agoraphobia (ref: No)	0.90	0.72	1.12	0.94	1	0.332
Posttraumatic stress disorder (ref: No)	0.98	0.77	1.24	0.04	1	0.845
Specific phobia (ref: No)	1.21	0.98	1.49	3.07	1	0.080
Social phobia (ref: No)	0.84	0.69	1.02	3.10	1	0.078
Substance use disorder (ref: No)	0.97	0.71	1.31	0.05	1	0.823
Treatment profiles						
General medical-only	0.46	0.38	0.54	75.98*	1	< 0.001
Psychiatrist-only	0.70	0.54	0.91	7.28*	1	0.007
Other mental health specialty-only	0.71	0.58	0.87	10.75*	1	0.001
Spiritual/healer-only	1.18	0.87	1.59	1.16	1	0.282
General medical with Psychiatrist	1.13	0.80	1.61	0.47	1	0.493
General medical with Other mental health specialty	1.18	0.92	1.51	1.63	1	0.201
Psychiatrist with Other mental health specialty	1.50	1.05	2.14	4.98*	1	0.026
General medical with Psychiatrist and Other mental health specialty	1.89	1.37	2.61	14.87*	1	< 0.001
Pooled *X*^2^ tests						
Mental disorders, *X*^2^_8_ (p-value)	18.53* (0.018)					
Treatment profiles, *X*^2^_7_ (p-value)	97.76* (< 0.001)					
Disorders and profiles, X^2^_15_ (p-value)	102.27* (< 0.001)					

^*^Significant at .05 level, two-sided test

Results shown are from the final model (see Additional file 1: Table A4 for details of the model-building process). Final model included survey dummy variables. The ORs associated with treatment profiles were centered to have a product of 0, allowing direct interpretation of each individual OR with the average in the total sample

^a^The General medical with Spiritual/healer treatment profile (n = 102) was dropped in the final model since it comprised a relatively small number of patients and made the modelling unstable, hence the sample size for the model is 3119

^b^Patient report of being helped 'a lot' by any type of provider seen

^c^High income was defined as greater than two times the within-country median per capita family income (i.e. income divided by number of family members), high-average income as 100–200% times the median, low-average as 50–100% of the median, and low income as less than 50% of the median

^d^In high-income countries, the high education category corresponded to a college degree, high-average to some post-secondary education without a college degree, low-average to secondary school graduation, and low to less than secondary education. These four categories comprised roughly equal sized groups. Thresholds in other countries were applied to achieve the same split

Treatment profile, in comparison, was a significant correlate (χ^2^_7_ = 97.76, p < 0.001). Specifically, the odds of being helped ‘a lot’ were significantly *lower* than average for the three most common profiles—General medical-only (χ^2^_1_ = 75.98, p < 0.001), Other mental health specialty-only (χ^2^_1_ = 10.75, p = 0.001) and Psychiatrist-only (χ^2^_1_ = 7.28, p = 0.007)—and significantly *higher* than average for the Psychiatrist with Other mental health specialty (χ^2^_1_ = 4.98, p = 0.026) and General medical with Psychiatrist and Other mental health specialty (χ^2^_1_ = 14.87, p < 0.001) profiles. It should be noted that the final model included only 8 of the 9 treatment profiles, as the General medical with Spiritual/healer profile was excluded because it comprised a relatively small number of patients and made the model unstable.

No significant interactions were found between sociodemographics and types of disorder, sociodemographics and treatment profiles, or types of disorders and treatment profiles with perceived helpfulness (see Additional file 1: Table A4 for details of the model-building process). We also examined the model separately for high-income countries (Additional file 1: Table A5) and low/middle-income countries (Additional file 1: Table A6), but results were very similar to those in the combined sample. A notable difference, though, was that in high-income countries, patients with 3 or more disorders had significantly higher relative-odds than others of being helped ‘a lot’ (OR = 1.62, 95% CI 1.03–2.55; χ^2^_1_ = 4.33, p = 0.037). Another was that, in low/middle-income countries, type of insurance was a significant correlate (χ^2^_4_ = 10.84, p = 0.028). Patients with state-funded insurance had more than twice the odds of being helped ‘a lot’, compared to those with no or unknown insurance (OR = 2.62, 95% CI 1.07–6.42).

## Discussion

### Key findings

We know of no previous study that has examined the perceived helpfulness of the service sectors seen by patients with mental and substance use disorders in as much detail, nor across such broad geographical scope, as we did here. We found that, across 17 countries combined, 58.2% of patients with 12-month mental and substance use disorders said that they were helped ‘a lot’ by the treatment profiles they used in the year prior to interview. Our key finding was that the odds of being helped ‘a lot’ were significantly higher (odds ratios [ORs] = 1.50–1.89) among the 12.9% of patients who used specialized multi-sector profiles involving both psychiatrists and other mental health specialists (with or without the general medical sector) than other patients, despite their high comorbidities. The lowest odds of being helped ‘a lot’ were found among patients who were seen only in the general medical, psychiatrist, or other mental health specialty sectors (ORs = 0.46–0.71). A few sociodemographic factors also influenced perceived helpfulness: female gender and older age were associated with increased odds of being helped ‘a lot’.

Our measure of perceived helpfulness represents the cumulative probability of being helped ‘a lot’, across all providers seen in the past year. This means that patients who received multi-sector care were more likely to have seen a greater number of providers than those who received single-sector care, and therefore to have had more opportunity to find a provider who helped them ‘a lot’. In this way, the current results are consistent with our earlier work on lifetime treatment, in which we found that patients who persisted in help-seeking efforts after earlier unhelpful treatments were significantly more likely than others eventually to obtain perceived helpful treatment [38–41]. Moreover, the most important predictors of between-patient differences in ever obtaining helpful treatment were the predictors of persistence in help-seeking after initial failures rather than predictors of any specific treatment encounter being helpful [38–41].

A further convergence of findings with our previous work on lifetime treatment [38–41] is that patients who received more specialized treatment tended to be the most likely to persist with help-seeking until they received treatment they perceived as helpful. We might have expected to find that the most specialized treatment profiles involving psychiatrists and other mental health professionals were used by patients with relatively more complex presentations that are more difficult to treat and who, therefore, may be *less* likely to be evaluated as helpful [24]. Indeed, we did find proportionally higher use of the multi-sector specialized profiles by patients with higher levels of comorbidity but, in the aggregate, the patients using these profiles were *more* likely to say they were helped ‘a lot’. This finding potentially extends prior associations between perceived helpfulness and specialized mental health sector use, by suggesting that this association is greater when treatment involves the specific combination of psychiatrists and non-medical mental health professionals [24, 26, 39]. This may be because these profiles are capable of delivering potentially effective treatments involving medication and psychological therapy, as needed. It could also reflect the provision of social interventions (including support with vocational, financial, social and housing needs) which are more likely to be needed by people with more complex presentations who, in this study, made up a relatively larger percentage of those who used the most specialized treatment profiles. Although the WMH surveys do not measure the content of interventions received in visits with specific health providers, this explanation is broadly consistent with evidence that improvements in functioning and social activities are indicators by which many service users judge their treatment to be effective [17]. Although we cannot know for certain that patients who used these multi-sector specialized profiles saw the different providers as part of shared care or multi-disciplinary care arrangements (as opposed to independent episodes of care), this finding might also suggest that collaborative or multidisciplinary treatment models which have been shown effective for people with severe and complex needs [42–45] are also viewed positively by patients.

Consistent with one previous study [46], perceived helpfulness was also high among patients seen in the two treatment profiles involving the Spiritual/healer sector. However, after adjusting for type of disorder, comorbidity and demographic factors in the multivariable regression model, the odds of being helped ‘a lot’ were no higher among those seen in the Spiritual/healer-only sector compared to the average across all treatment profiles. We were unable to include the General medical with Spiritual/healer profile in the regression model, due to the small number in this group.

Conversely, in the current study, the odds of being helped ‘a lot’ were halved among patients who were seen only in the general medical sector. This finding is consistent with previous reports of lower perceived helpfulness [24, 26] and greater likelihood of drop out from care [47] among patients seen in this treatment profile. This is of concern, given that the general medical only profile was used by more than one-third (35.4%) of patients in the current study (including 32.2% of those with 2 disorders and 27.3% of those with 3 or more disorders), and has been associated elsewhere with lower effective treatment coverage compared to the specialized mental health sector [26, 48–53]. Elsewhere, inadequate time for evaluation and treatment, lack of training, lack of specialized referral options, and preference for medication over psychotherapy among general practitioners have been identified as possible factors contributing to lower effective treatment coverage in the general medical sector [26, 54–56]. It was beyond the scope of this study to examine the correspondence between perceived helpfulness and effective treatment coverage, but this is an area for future focus.

With respect to sociodemographics, the positive association between older age and perceived helpfulness is consistent with other evidence that patients’ appraisals of mental health care and satisfaction with life in general improve with age [46, 57–61]. The negative association between male gender and perceived helpfulness has been reported in other samples limited to patients with diagnosed disorders [26], but not in samples of service users that include patients with and without 12-month disorders [14, 24, 61, 62]. Given that a significant proportion of people who use mental health services do not have a 12-month disorder but have other possible indicators of need (e.g., subthreshold problems, recent stressors or suicidality) [63], it could be that patterns of perceived helpfulness are different in the latter group.

In models stratified by country income group, having 3 or more disorders (high-income countries only) and state-funded health insurance (low/middle-income countries only) were associated with increased odds of being helped ‘a lot’. It may be that in high-income countries there are more enabling factors (e.g., supply of mental health specialists) that allow patients with more complex problems to persist with help-seeking until a helpful provider is found. In low/middle-income countries, arrangements established under state-funded or subsidized insurance (where available) may offer a more effective pathway to helpful providers than other forms of insurance.

### Limitations

The study has several limitations worth noting. First, the data were cross-sectional. Hence, we could not establish the timeline in relation to the receipt of specific services, various providers, and perceived helpfulness. For example, respondents were asked about the helpfulness of each type of provider seen in the past year, and from that we calculated the probability of being helped ‘a lot’ by *any* of the providers seen. We assumed that the multi-sector specialized profiles were more helpful because patients eventually received treatment from mental health specialists. However, the temporal ordering of the pathway through different providers could not be assessed in this study.

Second, we grouped patients according to the types of providers seen as well as their persistence in help-seeking across providers from different service sectors. Patients could not be randomly assigned to these conditions. Consequently, it is possible that various selection biases could play a role in the observed patterns. Indeed, we know from other research that a range of patient factors (e.g., self-selection), provider factors (e.g., referral bias) and system factors (e.g., provider supply, gatekeeper arrangements, and reimbursement policies) may determine where patients are treated and the extent to which they are able to persist in help-seeking [64, 65].

Finally, our measure of perceived helpfulness was based on a single question and we do not know how respondents interpreted being ‘helped’ or being helped ‘a lot’, nor how this global measure might align with more nuanced measures of the helpfulness of specific treatment components and foci. Moreover, current psychopathology or residual symptoms may influence respondents’ assessments regarding previous treatments. However, prior research has reported acceptable psychometric properties for single-global questions measuring mental and physical health [66–68] and our assessment procedures followed those of other studies with similar foci.

## Conclusions

Findings from this large, population sample are encouraging in that, among the 29.4% of people with a 12-month mental or substance use disorder who saw a provider in the past year, 58.2% said they were helped a lot. An additional 35.1% said they were helped ‘some’ or ‘a little', and only 6.7% were helped ‘not at all’. Patients who received specialized, multi-sector care were more likely to report that they were helped ‘a lot’. This result is consistent with previous research suggesting that persistence in help-seeking is associated with increased helpfulness of treatment. This analysis addresses a gap in knowledge about the patient’s perspective on the quality of mental health care as experienced in the real world.

## Supplementary Information

**Additional file 1: Table A1**. Coding of service sector combinations into 9 mutually exclusive treatment profiles, among respondents with 12-month DSM-IV disorders who reported 12-month use of providers for mental health (N=3221). **Table A2**. Associations of disorder types with perceived helpfulness, among respondents with 12-month DSM-IV disorders who reported 12-month use of providers for mental health (N=3221). **Table A3**. Associations of sociodemographics and disorder types with use of mutually exclusive treatment profiles, among respondents with 12-month DSM-IV disorders who reported 12-month use of providers for mental health. **Table A4**. Development of logistic regression models showing joint associations of sociodemographics, disorder types, and treatment profiles with perceived helpfulness (being helped 'a lot'), among respondents with 12-month DSM-IV disorders who reported 12-month use of providers for mental health, all countries combined (N=3119). **Table A5**. Development of logistic regression models showing joint associations of sociodemographics, disorder types, and treatment profiles with perceived helpfulness (being helped 'a lot'), among respondents with 12-month DSM-IV disorders who reported 12-month use of providers for mental health, high-income countries (N=2546). **Table A6**. Development of logistic regression models showing joint associations of sociodemographics, disorder types, and treatment profiles with perceived helpfulness (being helped 'a lot'), among respondents with 12-month DSM-IV disorders who reported 12-month use of providers for mental health, low/middle-income countries (N=573).

## Data Availability

Access to the cross-national World Mental Health (WMH) data is governed by the organizations funding and responsible for survey data collection in each country. These organizations made data available to the WMH consortium through restricted data sharing agreements that do not allow us to release the data to third parties. The exception is that the U.S. data are available for secondary analysis via the Inter-University Consortium for Political and Social Research (ICPSR), http://www.icpsr.umich.edu/icpsrweb/ICPSR/series/00527.

## Abbreviations

CIDI 3.0: Composite International Diagnostic Interview Version 3.0
DSM-IV: Diagnostic and Statistical Manual of Mental Disorders, Fourth Edition
OR: Odds ratio
SE: Standard error
WHO: World Health Organization
WMH: World Mental Health
