# Supplementary material for: Perceived helpfulness of service sectors used for mental and substance use disorders: Findings from the WHO World Mental Health Surveys
Source: Int J Ment Health Syst. 2022 Jan 29;16:6. doi: 10.1186/s13033-022-00516-z (PMC8800240; doi:10.1186/s13033-022-00516-z)
Supplement: Supplementary file 1 — Additional file 1: Table A1. Coding of service sector combinations into 9 mutually exclusive treatment profiles, among respondents with 12-month DSM-IV disorders who reported 12-month use of providers for mental health (N=3221). Table A2. Associations of disorder types with perceived helpfulness, among respondents with 12-month DSM-IV disorders who reported 12-month use of providers for mental health (N=3221). Table A3. Associations of sociodemographics and disorder types with use of mutually exclusive treatment profiles, among respondents with 12-month DSM-IV disorders who reported 12-month use of providers for mental health. Table A4. Development of logistic regression models showing joint associations of sociodemographics, disorder types, and treatment profiles with perceived helpfulness (being helped 'a lot'), among respondents with 12-month DSM-IV disorders who reported 12-month use of providers for mental health, all countries combined (N=3119). Table A5. Development of logistic regression models showing joint associations of sociodemographics, disorder types, and treatment profiles with perceived helpfulness (being helped 'a lot'), among respondents with 12-month DSM-IV disorders who reported 12-month use of providers for mental health, high-income countries (N=2546). Table A6. Development of logistic regression models showing joint associations of sociodemographics, disorder types, and treatment profiles with perceived helpfulness (being helped 'a lot'), among respondents with 12-month DSM-IV disorders who reported 12-month use of providers for mental health, low/middle-income countries (N=573). [file 13033_2022_516_MOESM1_ESM.docx]

**Additional File Tables**

| **Table A1. Coding of service sector combinations into 9 mutually exclusive treatment profiles, among respondents with 12-month DSM-IV disorders who reported 12-month use of providers for mental health (N=3221)** | | | | | |
| --- | --- | --- | --- | --- | --- |
|  | | | | | |
|  | **Service sector^a^ combination** | **%** | **SE** | **n** | **Recoded into treatment profile** |
| 9 treatment profiles that accounted for the majority of respondents (before rare combinations were recoded) | GM-only | 35.1 | 1.1 | 1111 | n/a |
|  | OMHS-only | 12.4 | 0.7 | 412 | n/a |
|  | PSY-only | 10.8 | 0.7 | 362 | n/a |
|  | GM + OMHS | 8.3 | 0.6 | 256 | n/a |
|  | SPH-only | 7.7 | 0.6 | 262 | n/a |
|  | GM + PSY + OMHS | 5.0 | 0.4 | 161 | n/a |
|  | PSY + OMHS | 5.0 | 0.4 | 160 | n/a |
|  | GM + PSY | 4.3 | 0.4 | 142 | n/a |
|  | GM + SPH | 3.3 | 0.4 | 97 | n/a |
|  | Subtotal | 91.8 |  |  |  |
| Rare combinations of service sectors that were recoded into the 9 treatment profiles | GM + OMHS + SPH | 1.6 | 0.3 | 46 | GM + OMHS |
|  | GM + PSY + OMHS + SPH | 1.4 | 0.3 | 40 | GM + PSY + OMHS |
|  | OMHS + SPH | 1.0 | 0.2 | 33 | OMHS-only |
|  | GM + PSY + SPH | 0.7 | 0.2 | 24 | GM + PSY |
|  | PSY + OMHS + SPH | 0.6 | 0.2 | 24 | PSY + OMHS |
|  | PSY + SPH | 0.6 | 0.2 | 19 | PSY-only |
|  | GM + PSY + OMHS + OHP | 0.5 | 0.1 | 13 | GM + PSY + OMHS |
|  | GM + OMHS + OHP | 0.4 | 0.1 | 15 | GM + OMHS |
|  | OMHS + OHP | 0.3 | 0.1 | 10 | OMHS-only |
|  | PSY + OMHS + OHP | 0.2 | 0.1 | 6 | PSY + OMHS |
|  | PSY + OHP | 0.2 | 0.1 | 4 | PSY-only |
|  | GM + PSY + OMHS + OHP + SPH | 0.1 | 0.1 | 6 | GM + PSY + OMHS |
|  | GM + OHP + SPH | 0.1 | 0.1 | 5 | GM + SPH |
|  | PSY + OMHS + OHP + SPH | 0.1 | 0.1 | 2 | PSY + OMHS |
|  | PSY + GM + OHP | 0.1 | 0.1 | 3 | GM + PSY |
|  | OHP + SPH | 0.1 | 0.1 | 3 | SPH-only |
|  | OMHS + OHP + SPH | 0.1 | 0.0 | 3 | OMHS-only |
|  | PSY + OHP + SPH | 0.0 | 0.0 | 1 | PSY-only |
|  | GM + PSY + OHP + SPH | 0.0 | 0.0 | 1 | GM + PSY |
|  | Subtotal | 8.2 |  |  |  |
| Total |  | 100.0 |  | 3221 |  |
| Abbreviations: n/a, not applicable. | | | | | |
| *^a^* Service sectors: GM - General medical (general practitioner/primary care doctor or other medical doctor); PSY - Psychiatrist; OMHS - Other mental health specialty (psychologist, any other mental health professional in any setting, a social worker or counselor in a mental health specialized setting); OHP - Other health provider (social worker or counselor in a human services setting, or a non-medical health professional); and SPH - Spiritual/healer (spiritual advisor or healer). | | | | | |

| **Table A2. Associations of disorder types with perceived helpfulness, among respondents with 12-month DSM-IV disorders who reported 12-month use of providers for mental health (N=3221)** | | | | | | | | | | | | |
| --- | --- | --- | --- | --- | --- | --- | --- | --- | --- | --- | --- | --- |
|  | | | | | | | | | | | | |
|  | **Perceived helpfulness^a^** | | | | | | | |  | **Chi-Square Test for Equal Proportions** | | |
|  | **‘A lot’** | | **‘Some’** | | **‘A little’** | | **‘Not at all’** | |  |  |  |  |
|  | **row %** | **SE** | **row %** | **SE** | **row %** | **SE** | **row %** | **SE** | **n** | ***X*^2^** | **df** | **p-value** |
| 12-month DSM-IV disorders |  |  |  |  |  |  |  |  |  |  |  |  |
| Major depressive disorder | 57.4 | 1.6 | 24.7 | 1.4 | 11.0 | 1.1 | 6.9 | 0.7 | 1470 | 541.77* | 3 | <0.001 |
| Bipolar disorder | 55.2 | 3.6 | 26.8 | 3.1 | 13.6 | 2.3 | 4.4 | 1.4 | 273 | 89.43* | 3 | <0.001 |
| Generalized anxiety disorder | 57.3 | 2.9 | 23.5 | 2.4 | 11.0 | 2.2 | 8.2 | 1.4 | 468 | 164.74* | 3 | <0.001 |
| Panic disorder/Agoraphobia | 58.6 | 2.2 | 25.4 | 2.0 | 9.6 | 1.2 | 6.3 | 1.0 | 645 | 269.22* | 3 | <0.001 |
| Posttraumatic stress disorder | 61.4 | 2.5 | 23.1 | 2.1 | 10.6 | 1.4 | 4.9 | 1.7 | 519 | 237.67* | 3 | <0.001 |
| Specific phobia | 62.3 | 1.7 | 19.9 | 1.3 | 11.1 | 1.1 | 6.7 | 0.9 | 1085 | 490.77* | 3 | <0.001 |
| Social phobia | 57.4 | 2.0 | 25.2 | 2.0 | 11.2 | 1.2 | 6.2 | 0.9 | 764 | 293.87* | 3 | <0.001 |
| Substance use disorder | 54.0 | 3.1 | 25.4 | 2.7 | 14.0 | 2.3 | 6.7 | 1.5 | 370 | 169.19* | 3 | <0.001 |
| Number of 12-month disorders |  |  |  |  |  |  |  |  |  |  |  |  |
| Exactly 1 | 58.1 | 1.3 | 25.2 | 1.1 | 9.8 | 0.8 | 7.0 | 0.8 | 1783 | 8.04 | 6 | 0.240 |
| Exactly 2 | 56.7 | 2.0 | 23.9 | 1.8 | 13.8 | 1.6 | 5.6 | 0.8 | 811 |  |  |  |
| 3 or more | 60.5 | 2.2 | 21.9 | 1.8 | 10.4 | 1.3 | 7.2 | 1.1 | 627 |  |  |  |
| * Significant at .05 level, two-sided test. | | | | | | | | | | | | |
| *^a^* The maximum rating of how much the patient said they were helped by any type of provider seen. | | | | | | | | | | | | |

| **Table A3. Associations of sociodemographics and disorder types with use of mutually exclusive treatment profiles, among respondents with 12-month DSM-IV disorders who reported 12-month use of providers for mental health** | | | | | | | | | | | | | | | | | | | |
| --- | --- | --- | --- | --- | --- | --- | --- | --- | --- | --- | --- | --- | --- | --- | --- | --- | --- | --- | --- |
|  | | | | | | | | | | | | | | | | | | | |
|  | **Treatment profiles** | | | | | | | | | | | | | | | | | |  |
|  | **General medical-only** | | **Psychiatrist-only** | | **Other mental health specialty-only** | | **Spiritual/healer-only** | | **General medical with Psychiatrist** | | **General medical with Other mental health specialty** | | **General medical with Spiritual/healer** | | **Psychiatrist with Other mental health specialty** | | **General medical with Psychiatrist and Other mental health specialty** | |  |
|  | **(n=1111)** | | **(n=386)** | | **(n=458)** | | **(n=265)** | | **(n=170)** | | **(n=317)** | | **(n=102)** | | **(n=192)** | | **(n=220)** | |  |
|  | **%** | **SE** | **%** | **SE** | **%** | **SE** | **%** | **SE** | **%** | **SE** | **%** | **SE** | **%** | **SE** | **%** | **SE** | **%** | **SE** | **n** |
| Gender |  |  |  |  |  |  |  |  |  |  |  |  |  |  |  |  |  |  |  |
| Male | 30.7 | 2.0 | 14.4 | 1.3 | 15.4 | 1.2 | 6.6 | 0.9 | 6.0 | 1.0 | 10.4 | 1.1 | 2.1 | 0.6 | 6.3 | 1.0 | 8.3 | 1.1 | 2308 |
| Female | 37.4 | 1.2 | 10.3 | 0.8 | 13.0 | 0.9 | 8.4 | 0.7 | 4.7 | 0.5 | 10.2 | 0.8 | 3.8 | 0.5 | 5.8 | 0.5 | 6.5 | 0.6 | 913 |
| *X*^2^_1_ (p-value) | 8.30* (0.004) | | 6.98* (0.008) | | 2.12 (0.146) | | 1.93 (0.165) | | 1.51 (0.219) | | 0.01 (0.926) | | 4.25* (0.039) | | 0.18 (0.671) | | 2.08 (0.149) | |  |
| Age at interview (years) |  |  |  |  |  |  |  |  |  |  |  |  |  |  |  |  |  |  |  |
| ≤34 years | 26.7 | 2.1 | 8.9 | 1.2 | 22.2 | 1.9 | 10.3 | 1.4 | 3.9 | 0.8 | 13.8 | 1.6 | 2.4 | 0.8 | 6.8 | 1.2 | 5.1 | 1.2 | 687 |
| 35-49 | 33.3 | 1.6 | 11.1 | 1.0 | 14.7 | 1.1 | 7.7 | 0.7 | 5.6 | 0.8 | 10.6 | 1.0 | 3.1 | 0.6 | 6.7 | 0.9 | 7.3 | 0.9 | 1227 |
| 50-64 | 39.1 | 1.8 | 15.5 | 1.4 | 8.3 | 1.0 | 6.0 | 0.8 | 5.4 | 0.8 | 8.0 | 0.9 | 4.4 | 0.9 | 4.8 | 0.7 | 8.6 | 1.1 | 967 |
| ≥65 | 55.1 | 3.1 | 9.1 | 1.8 | 3.5 | 1.5 | 6.5 | 1.7 | 6.0 | 1.2 | 6.1 | 1.5 | 2.9 | 1.0 | 4.0 | 1.3 | 6.8 | 1.6 | 340 |
| *X*^2^_3_ (p-value) | 51.25* (<0.001) | | 13.02* (0.005) | | 59.61* (<0.001) | | 7.34 (0.062) | | 2.28 (0.516) | | 13.21* (0.004) | | 3.58 (0.311) | | 3.97 (0.265) | | 4.80 (0.187) | |  |
| Marital status |  |  |  |  |  |  |  |  |  |  |  |  |  |  |  |  |  |  |  |
| Married/cohabitating | 40.3 | 1.6 | 12.0 | 0.9 | 11.8 | 0.9 | 8.1 | 0.8 | 6.1 | 0.7 | 8.3 | 0.8 | 3.4 | 0.6 | 4.3 | 0.5 | 5.9 | 0.7 | 1632 |
| Separated/widowed/divorced | 35.1 | 2.0 | 11.3 | 1.5 | 11.0 | 1.3 | 8.0 | 1.2 | 4.1 | 0.8 | 11.2 | 1.2 | 3.5 | 0.8 | 6.6 | 1.1 | 9.2 | 1.3 | 807 |
| Never married | 25.3 | 1.7 | 10.9 | 1.2 | 20.0 | 1.8 | 7.1 | 1.0 | 4.0 | 0.8 | 13.5 | 1.4 | 2.9 | 0.8 | 8.6 | 1.3 | 7.7 | 1.2 | 782 |
| *X*^2^_2_ (p-value) | 33.34* (<0.001) | | 0.26 (0.879) | | 22.29* (<0.001) | | 0.54 (0.763) | | 4.06 (0.131) | | 10.16* (0.006) | | 0.26 (0.877) | | 11.59* (0.003) | | 5.23 (0.073) | |  |
| Family income^a^ |  |  |  |  |  |  |  |  |  |  |  |  |  |  |  |  |  |  |  |
| Low | 31.7 | 1.9 | 12.7 | 1.2 | 12.7 | 1.3 | 7.5 | 1.1 | 5.8 | 0.8 | 11.2 | 1.3 | 3.5 | 0.8 | 7.6 | 1.2 | 7.3 | 1.0 | 1089 |
| Low-average | 35.9 | 2.1 | 9.5 | 1.5 | 13.3 | 1.5 | 9.9 | 1.3 | 3.5 | 0.7 | 10.2 | 1.1 | 4.5 | 0.9 | 5.5 | 1.0 | 7.7 | 1.0 | 802 |
| High-average | 33.9 | 2.2 | 12.7 | 1.6 | 13.9 | 1.4 | 9.5 | 1.2 | 6.2 | 1.1 | 9.6 | 1.4 | 2.2 | 0.7 | 5.4 | 0.9 | 6.8 | 1.3 | 704 |
| High | 33.2 | 2.2 | 14.7 | 1.8 | 17.9 | 2.0 | 5.8 | 1.2 | 3.8 | 0.8 | 11.1 | 1.4 | 3.0 | 1.0 | 6.7 | 1.1 | 3.8 | 0.8 | 626 |
| *X*^2^_3_ (p-value) | 1.72 (0.632) | | 6.18 (0.103) | | 6.97 (0.073) | | 8.66* (0.034) | | 6.18 (0.103) | | 0.48 (0.923) | | 3.74 (0.291) | | 0.87 (0.832) | | 7.81 (0.050) | |  |
| Education^b^ |  |  |  |  |  |  |  |  |  |  |  |  |  |  |  |  |  |  |  |
| Low | 43.6 | 2.5 | 12.0 | 1.6 | 10.9 | 1.5 | 6.3 | 1.0 | 6.2 | 1.1 | 7.4 | 1.1 | 1.8 | 0.6 | 6.1 | 1.2 | 5.7 | 1.1 | 597 |
| Low-average | 37.6 | 2.2 | 12.2 | 1.3 | 11.3 | 1.3 | 8.7 | 1.3 | 4.3 | 0.9 | 10.0 | 1.6 | 3.8 | 0.9 | 5.2 | 1.0 | 6.8 | 1.0 | 726 |
| High-average | 34.4 | 1.8 | 11.2 | 1.1 | 14.3 | 1.1 | 7.5 | 0.9 | 5.7 | 0.7 | 10.8 | 1.1 | 3.4 | 0.7 | 5.6 | 0.9 | 7.3 | 0.9 | 1121 |
| High | 28.4 | 2.0 | 11.3 | 1.3 | 17.3 | 1.7 | 8.5 | 1.2 | 4.1 | 0.8 | 11.7 | 1.2 | 3.7 | 0.9 | 7.1 | 1.1 | 7.9 | 1.3 | 777 |
| *X*^2^_3_ (p-value) | 20.54* (<0.001) | | 0.39 (0.941) | | 9.33* (0.025) | | 1.85 (0.605) | | 2.88 (0.411) | | 4.08 (0.253) | | 3.03 (0.387) | | 1.82 (0.611) | | 1.51 (0.679) | |  |

*Continued over.*

| **Table A3 continued. Associations of sociodemographics and disorder types with use of mutually exclusive treatment profiles, among respondents with 12-month DSM-IV disorders who reported 12-month use of providers for mental health** | | | | | | | | | | | | | | | | | | | |
| --- | --- | --- | --- | --- | --- | --- | --- | --- | --- | --- | --- | --- | --- | --- | --- | --- | --- | --- | --- |
|  | | | | | | | | | | | | | | | | | | | |
|  | **Treatment profiles** | | | | | | | | | | | | | | | | | |  |
|  | **General medical-only** | | **Psychiatrist-only** | | **Other mental health specialty-only** | | **Spiritual/healer-only** | | **General medical with Psychiatrist** | | **General medical with Other mental health specialty** | | **General medical with Spiritual/healer** | | **Psychiatrist with Other mental health specialty** | | **General medical with Psychiatrist and Other mental health specialty** | |  |
|  | **(n=1111)** | | **(n=386)** | | **(n=458)** | | **(n=265)** | | **(n=170)** | | **(n=317)** | | **(n=102)** | | **(n=192)** | | **(n=220)** | |  |
|  | **%** | **SE** | **%** | **SE** | **%** | **SE** | **%** | **SE** | **%** | **SE** | **%** | **SE** | **%** | **SE** | **%** | **SE** | **%** | **SE** | **n** |
| Employment |  |  |  |  |  |  |  |  |  |  |  |  |  |  |  |  |  |  |  |
| Homemaker | 36.7 | 2.6 | 16.4 | 2.0 | 12.0 | 1.6 | 10.0 | 1.7 | 4.5 | 1.0 | 8.3 | 1.4 | 3.8 | 1.1 | 3.6 | 0.7 | 4.8 | 1.1 | 468 |
| Retired | 53.0 | 3.1 | 14.6 | 2.4 | 3.5 | 1.7 | 3.2 | 0.8 | 7.2 | 1.4 | 5.6 | 1.3 | 2.4 | 1.0 | 3.9 | 1.3 | 6.6 | 1.6 | 316 |
| Student | 22.5 | 4.7 | 6.6 | 2.4 | 23.4 | 4.2 | 9.2 | 2.8 | 0.9 | 1.4 | 22.0 | 4.5 | 3.2 | 1.6 | 6.9 | 2.5 | 5.2 | 2.0 | 138 |
| Working | 35.2 | 1.5 | 9.7 | 0.9 | 15.5 | 1.1 | 8.3 | 0.7 | 5.3 | 0.7 | 11.6 | 0.8 | 3.6 | 0.6 | 5.0 | 0.6 | 5.7 | 0.7 | 1797 |
| Other | 28.5 | 2.5 | 14.6 | 1.8 | 10.4 | 1.5 | 5.8 | 1.1 | 5.4 | 1.1 | 5.6 | 1.0 | 2.1 | 0.7 | 12.4 | 1.9 | 15.2 | 1.9 | 502 |
| *X*^2^_4_ (p-value) | 37.97* (<0.001) | | 15.78* (0.003) | | 26.55* (<0.001) | | 7.89 (0.096) | | 6.00 (0.199) | | 31.78* (<0.001) | | 2.81 (0.590) | | 28.16* (<0.001) | | 36.21* (<0.001) | |  |
| Insurance |  |  |  |  |  |  |  |  |  |  |  |  |  |  |  |  |  |  |  |
| None or unknown | 28.1 | 3.8 | 11.5 | 2.5 | 24.8 | 2.7 | 12.5 | 2.4 | 1.4 | 1.4 | 5.0 | 2.1 | 2.2 | 1.3 | 8.1 | 2.3 | 6.5 | 1.8 | 1459 |
| State funded coverage or subsidized insurance | 31.7 | 2.2 | 20.3 | 1.8 | 9.1 | 1.2 | 7.3 | 1.3 | 8.4 | 1.2 | 5.1 | 0.9 | 1.3 | 0.5 | 10.5 | 1.5 | 6.4 | 0.9 | 624 |
| Insurance through employment or national   social security | 24.8 | 2.8 | 19.3 | 3.2 | 21.7 | 3.5 | 10.2 | 2.4 | 4.2 | 1.4 | 6.8 | 2.0 | 1.6 | 0.9 | 6.5 | 1.9 | 4.9 | 1.2 | 266 |
| Direct private/optional insurance | 37.4 | 6.7 | 15.4 | 4.4 | 21.2 | 5.5 | 0.6 | 0.6 | 0.6 | 0.6 | 7.8 | 3.8 | 0.0 | 0.0 | 12.6 | 4.5 | 4.4 | 1.0 | 65 |
| Other | 29.8 | 1.6 | 15.7 | 1.7 | 15.6 | 1.6 | 7.1 | 1.0 | 4.6 | 0.8 | 8.3 | 1.5 | 3.9 | 0.9 | 7.5 | 1.0 | 7.4 | 1.1 | 807 |
| *X*^2^_4_ (p-value) | 28.60* (<0.001) | | 83.76* (<0.001) | | 18.14* (0.001) | | 4.62 (0.328) | | 10.51* (0.033) | | 26.93* (<0.001) | | 9.38 (0.052) | | 30.92* (<0.001) | | 2.25 (0.691) | |  |
| 12-month DSM-IV disorders |  |  |  |  |  |  |  |  |  |  |  |  |  |  |  |  |  |  |  |
| Major depressive disorder | 34.6 | 1.5 | 13.6 | 1.0 | 11.8 | 0.9 | 5.8 | 0.6 | 5.7 | 0.7 | 12.2 | 1.0 | 3.4 | 0.6 | 6.5 | 0.8 | 6.5 | 0.7 | 1470 |
| *X*^2^_1_ (p-value) | 0.29 (0.588) | | 5.84* (0.016) | | 5.03* (0.025) | | 8.54* (0.004) | | 1.25 (0.264) | | 6.37* (0.012) | | 0.04 (0.835) | | 0.74 (0.390) | | 0.68 (0.409) | |  |
| Bipolar disorder | 23.7 | 3.2 | 15.2 | 3.3 | 11.0 | 2.1 | 6.1 | 1.4 | 6.7 | 1.6 | 10.5 | 2.1 | 0.8 | 0.6 | 6.9 | 1.4 | 19.1 | 3.4 | 273 |
| *X*^2^_1_ (p-value) | 9.66* (0.002) | | 2.09 (0.149) | | 1.05 (0.310) | | 0.64 (0.424) | | 0.91 (0.341) | | 0.008 (0.930) | | 3.19 (0.074) | | 0.28 (0.598) | | 36.47* (<0.001) | |  |
| Generalized anxiety disorder | 33.6 | 2.7 | 11.9 | 2.2 | 10.1 | 1.6 | 10.4 | 1.8 | 7.1 | 1.3 | 10.2 | 1.5 | 3.7 | 1.0 | 4.4 | 1.1 | 8.6 | 1.6 | 468 |
| *X*^2^_1_ (p-value) | 0.39 (0.534) | | 0.03 (0.856) | | 3.52 (0.061) | | 3.06 (0.080) | | 2.64 (0.104) | | 0.003 (0.955) | | 0.97 (0.658) | | 1.38 (0.241) | | 1.15 (0.283) | |  |
| Panic disorder/Agoraphobia | 31.8 | 1.8 | 10.5 | 1.5 | 7.2 | 1.2 | 8.1 | 1.0 | 6.8 | 1.2 | 11.9 | 1.4 | 3.2 | 1.0 | 7.0 | 1.2 | 13.4 | 1.6 | 645 |
| *X*^2^_1_ (p-value) | 2.51 (0.113) | | 0.59 (0.441) | | 17.97* (<0.001) | | 0.08 (0.781) | | 2.93 (0.087) | | 1.41 (0.234) | | 0.001 (0.971) | | 1.06 (0.303) | | 30.54* (<0.001) | |  |
| Posttraumatic stress disorder | 30.1 | 2.5 | 7.3 | 1.3 | 12.9 | 1.8 | 9.2 | 1.8 | 4.7 | 0.9 | 13.8 | 1.6 | 4.1 | 1.4 | 8.1 | 1.4 | 9.8 | 1.4 | 519 |
| *X*^2^_1_ (p-value) | 4.25* (0.039) | | 6.43* (0.011) | | 0.24 (0.627) | | 1.05 (0.306) | | 0.12 (0.728) | | 5.02* (0.025) | | 0.79 (0.374) | | 2.97 (0.085) | | 4.13* (0.042) | |  |
| Specific phobia | 36.8 | 1.8 | 11.6 | 1.1 | 11.9 | 1.1 | 8.8 | 1.1 | 5.7 | 0.8 | 8.2 | 0.8 | 3.7 | 0.8 | 5.2 | 0.8 | 8.1 | 1.1 | 1085 |
| *X*^2^_1_ (p-value) | 0.99 (0.319) | | 0.001 (0.979) | | 2.72 (0.099) | | 1.37 (0.242) | | 0.68 (0.409) | | 4.56* (0.033) | | 0.64 (0.423) | | 0.93 (0.336) | | 1.58 (0.210) | |  |

*Continued over.*

| **Table A3 continued. Associations of sociodemographics and disorder types with use of mutually exclusive treatment profiles, among respondents with 12-month DSM-IV disorders who reported 12-month use of providers for mental health** | | | | | | | | | | | | | | | | | | | |
| --- | --- | --- | --- | --- | --- | --- | --- | --- | --- | --- | --- | --- | --- | --- | --- | --- | --- | --- | --- |
|  | | | | | | | | | | | | | | | | | | | |
|  | **Treatment profiles** | | | | | | | | | | | | | | | | | |  |
|  | **General medical-only** | | **Psychiatrist-only** | | **Other mental health specialty-only** | | **Spiritual/healer-only** | | **General medical with Psychiatrist** | | **General medical with Other mental health specialty** | | **General medical with Spiritual/healer** | | **Psychiatrist with Other mental health specialty** | | **General medical with Psychiatrist and Other mental health specialty** | |  |
|  | **(n=1111)** | | **(n=386)** | | **(n=458)** | | **(n=265)** | | **(n=170)** | | **(n=317)** | | **(n=102)** | | **(n=192)** | | **(n=220)** | |  |
|  | **%** | **SE** | **%** | **SE** | **%** | **SE** | **%** | **SE** | **%** | **SE** | **%** | **SE** | **%** | **SE** | **%** | **SE** | **%** | **SE** | **n** |
| Social phobia | 30.1 | 1.8 | 11.6 | 1.5 | 11.0 | 1.2 | 7.5 | 1.0 | 6.9 | 1.1 | 12.6 | 1.3 | 2.8 | 0.7 | 6.5 | 0.9 | 10.9 | 1.6 | 764 |
| *X*^2^_1_ (p-value) | 7.06* (0.008) | | 0.0003 (0.987) | | 3.98* (0.046) | | 0.06 (0.809) | | 4.25* (0.039) | | 3.59 (0.058) | | 0.42 (0.517) | | 0.39 (0.534) | | 13.94* (<0.001) | |  |
| Substance use disorder | 22.8 | 2.8 | 13.2 | 2.4 | 20.3 | 2.5 | 5.9 | 1.5 | 4.3 | 1.2 | 14.3 | 2.1 | 3.1 | 1.4 | 7.8 | 1.8 | 8.2 | 1.3 | 370 |
| *X*^2^_1_ (p-value) | 18.66* (<0.001) | | 0.73 (0.393) | | 9.86* (0.002) | | 1.32 (0.251) | | 0.33 (0.566) | | 4.97* (0.026) | | 0.03 (0.866) | | 1.65 (0.199) | | 0.56 (0.452) | |  |
| Number of 12-month disorders |  |  |  |  |  |  |  |  |  |  |  |  |  |  |  |  |  |  |  |
| Exactly 1 | 39.30 | 1.60 | 11.00 | 0.80 | 16.80 | 1.10 | 8.00 | 0.80 | 3.60 | 0.50 | 8.10 | 0.80 | 3.10 | 0.50 | 5.50 | 0.60 | 4.70 | 0.60 | 1783 |
| Exactly 2 | 32.30 | 1.90 | 12.40 | 1.30 | 11.40 | 1.20 | 7.70 | 0.90 | 6.90 | 1.00 | 13.70 | 1.40 | 3.70 | 0.90 | 5.50 | 0.90 | 6.50 | 1.10 | 811 |
| 3 or more | 27.30 | 2.00 | 12.20 | 1.80 | 8.00 | 1.10 | 7.50 | 1.20 | 7.10 | 1.10 | 12.40 | 1.30 | 3.10 | 1.00 | 7.80 | 1.20 | 14.70 | 1.80 | 627 |
| *X*^2^_2_ (p-value) | 19.81* (<0.001) | | 0.77 (0.679) | | 20.92* (<0.001) | | 0.11 (0.948) | | 11.40* (0.003) | | 13.51* (0.001) | | 0.32 (0.851) | | 2.89 (0.236) | | 42.49* (<0.001) | |  |
| Any 12-month disorder | 35.2 | 1.1 | 11.6 | 0.7 | 13.8 | 0.7 | 7.8 | 0.6 | 5.1 | 0.4 | 10.3 | 0.6 | 3.3 | 0.4 | 5.9 | 0.5 | 7.0 | 0.6 | 3221 |
| **Pooled *X*^2^ tests** |  | | | | | | | | | | | | | | | | | | |
| Gender, *X*^2^_8_ (p-value) | 22.81* (0.004) | | | | | | | | | | | | | | | | | | |
| Age at interview, *X*^2^_24_ (p-value) | 128.55* (<0.001) | | | | | | | | | | | | | | | | | | |
| Marital status, *X*^2^_16_ (p-value) | 70.54* (<0.001) | | | | | | | | | | | | | | | | | | |
| Family income, *X*^2^_24_ (p-value) | 38.57* (0.030) | | | | | | | | | | | | | | | | | | |
| Education, *X*^2^_24_ (p-value) | 35.85 (0.057) | | | | | | | | | | | | | | | | | | |
| Employment, *X*^2^_32_ (p-value) | 165.79* (<0.001) | | | | | | | | | | | | | | | | | | |
| Insurance, *X*^2^_32_ (p-value) | 28.77* (<0.001) | | | | | | | | | | | | | | | | | | |
| Mental disorders, *X*^2^_8_ (p-value) | 186.85* (<0.001) | | | | | | | | | | | | | | | | | | |
| Number of disorders, *X*^2^_16_ (p-value) | 91.12* (<0.001) | | | | | | | | | | | | | | | | | | |
| * Significant at .05 level, two-sided test. | | | | | | | | | | | | | | | | | | | |
| *^a^* High income was defined as greater than two times the within-country median per capita family income (i.e., income divided by number of family members), high-average income as 100-200% times the median, low-average as 50–100% of the median, and low income as less than 50% of the median. | | | | | | | | | | | | | | | | | | | |
| *^b^* In high-income countries, the high education category corresponded to a college degree, high-average to some post-secondary education without a college degree, low-average to secondary school graduation, and low to less than secondary education. These four categories comprised roughly equal sized groups. Thresholds in other countries were applied to achieve the same split. | | | | | | | | | | | | | | | | | | | |

| **Table A4. Development of logistic regression models showing joint associations of sociodemographics, disorder types, and treatment profiles with perceived helpfulness (being helped 'a lot'), among respondents with 12-month DSM-IV disorders who reported 12-month use of providers for mental health, all countries combined (N=3119)^a^** | | | | | | | | | | | | | | | |
| --- | --- | --- | --- | --- | --- | --- | --- | --- | --- | --- | --- | --- | --- | --- | --- |
|  | | | | | | | | | | | | | | | |
|  | **Perceived helpfulness (being helped 'a lot')^b^** | | | | | | | | | | | | | | |
|  | **Model 1a:** Original pooled model | | | **Model 1b:** Pooled model including 3+ disorders | | | **Model 2:** lnclude the original left out category General medical-only), all included dummies subtracted by the original 7th dummy (General medical with Psychiatrist and Other mental health specialty) | | | **Model 3:** lnclude the original 7 dummies, all included dummies subtracted by the left out category (General medical-only) | | | **Final Model:** with pooled ORs from Model 2 and Model 3 | | |
|  | **OR** | **95% CI** | | **OR** | **95% CI** | | **OR** | **95% CI** | | **OR** | **95% CI** | | **OR** | **95% CI** | |
| Gender (ref: Female) | reference |  |  | reference |  |  | reference |  |  | reference |  |  | reference |  |  |
| Male | 0.76 | 0.60 | 0.96 | 0.76 | 0.60 | 0.96 | 0.76 | 0.60 | 0.96 | 0.76 | 0.60 | 0.96 | 0.76 | 0.60 | 0.96 |
| *X*^2^_1_ (p-value) | 5.40* (0.020) | | | 5.36* (0.021) | | | 5.40* (0.020) | | | 5.40* (0.020) | | | 5.40* (0.020) | | |
| Age at interview (years) (ref: ≥65) | reference |  |  | reference |  |  | reference |  |  | reference |  |  | reference |  |  |
| ≤34 years | 0.61 | 0.36 | 1.02 | 0.61 | 0.36 | 1.03 | 0.61 | 0.36 | 1.02 | 0.61 | 0.36 | 1.02 | 0.61 | 0.36 | 1.02 |
| 35-49 | 0.77 | 0.49 | 1.21 | 0.78 | 0.50 | 1.22 | 0.77 | 0.49 | 1.21 | 0.77 | 0.49 | 1.21 | 0.77 | 0.49 | 1.21 |
| 50-64 | 1.13 | 0.74 | 1.73 | 1.14 | 0.74 | 1.75 | 1.13 | 0.74 | 1.73 | 1.13 | 0.74 | 1.73 | 1.13 | 0.74 | 1.73 |
| *X*^2^_3_ (p-value) | 17.34* (0.001) | | | 17.59* (0.001) | | | 17.34* (0.001) | | | 17.34* (0.001) | | | 17.34* (0.001) | | |
| Marital status (ref: Married/cohabitating) | reference |  |  | reference |  |  | reference |  |  | reference |  |  | reference |  |  |
| Separated/widowed/divorced | 0.94 | 0.76 | 1.17 | 0.95 | 0.76 | 1.18 | 0.94 | 0.76 | 1.17 | 0.94 | 0.76 | 1.17 | 0.94 | 0.76 | 1.17 |
| Never married | 1.04 | 0.81 | 1.34 | 1.04 | 0.81 | 1.34 | 1.04 | 0.81 | 1.34 | 1.04 | 0.81 | 1.34 | 1.04 | 0.81 | 1.34 |
| *X*^2^_2_ (p-value) | 0.54 (0.764) | | | 0.46 (0.796) | | | 0.54 (0.764) | | | 0.54 (0.764) | | | 0.54 (0.764) | | |
| Family income^c^ (ref: High) | reference |  |  | reference |  |  | reference |  |  | reference |  |  | reference |  |  |
| Low | 0.87 | 0.66 | 1.14 | 0.87 | 0.67 | 1.15 | 0.87 | 0.66 | 1.14 | 0.87 | 0.66 | 1.14 | 0.87 | 0.66 | 1.14 |
| Low-average | 0.89 | 0.70 | 1.13 | 0.88 | 0.70 | 1.12 | 0.89 | 0.70 | 1.13 | 0.89 | 0.70 | 1.13 | 0.89 | 0.70 | 1.13 |
| High-average | 0.87 | 0.67 | 1.13 | 0.87 | 0.67 | 1.13 | 0.87 | 0.67 | 1.13 | 0.87 | 0.67 | 1.13 | 0.87 | 0.67 | 1.13 |
| *X*^2^_3_ (p-value) | 1.54 (0.672) | | | 1.53 (0.677) | | | 1.54 (0.672) | | | 1.54 (0.672) | | | 1.54 (0.672) | | |
| Education^d^ (ref: High) | reference |  |  | reference |  |  | reference |  |  | reference |  |  | reference |  |  |
| Low | 1.04 | 0.77 | 1.40 | 1.04 | 0.78 | 1.40 | 1.04 | 0.77 | 1.40 | 1.04 | 0.77 | 1.40 | 1.04 | 0.77 | 1.40 |
| Low-average | 0.98 | 0.75 | 1.29 | 0.98 | 0.74 | 1.28 | 0.98 | 0.75 | 1.29 | 0.98 | 0.75 | 1.29 | 0.98 | 0.75 | 1.29 |
| High-average | 0.89 | 0.70 | 1.12 | 0.89 | 0.71 | 1.12 | 0.89 | 0.70 | 1.12 | 0.89 | 0.70 | 1.12 | 0.89 | 0.70 | 1.12 |
| *X*^2^_3_ (p-value) | 1.55 (0.671) | | | 1.51 (0.680) | | | 1.55 (0.671) | | | 1.55 (0.671) | | | 1.55 (0.671) | | |
| Employment (ref: Working) | reference |  |  | reference |  |  | reference |  |  | reference |  |  | reference |  |  |
| Homemaker | 0.85 | 0.63 | 1.15 | 0.85 | 0.63 | 1.15 | 0.85 | 0.63 | 1.15 | 0.85 | 0.63 | 1.15 | 0.85 | 0.63 | 1.15 |
| Retired | 1.07 | 0.72 | 1.60 | 1.07 | 0.72 | 1.60 | 1.07 | 0.72 | 1.60 | 1.07 | 0.72 | 1.60 | 1.07 | 0.72 | 1.60 |
| Student | 0.79 | 0.50 | 1.24 | 0.79 | 0.50 | 1.24 | 0.79 | 0.50 | 1.24 | 0.79 | 0.50 | 1.24 | 0.79 | 0.50 | 1.24 |
| Other | 0.73 | 0.56 | 0.95 | 0.73 | 0.56 | 0.95 | 0.73 | 0.56 | 0.95 | 0.73 | 0.56 | 0.95 | 0.73 | 0.56 | 0.95 |
| *X*^2^_4_ (p-value) | 6.70 (0.153) | | | 6.66 (0.155) | | | 6.70 (0.153) | | | 6.70 (0.153) | | | 6.70 (0.153) | | |

*Continued over.*

| **Table A4 continued. Development of logistic regression models showing joint associations of sociodemographics, disorder types, and treatment profiles with perceived helpfulness (being helped 'a lot'), among respondents with 12-month DSM-IV disorders who reported 12-month use of providers for mental health, all countries combined (N=3119)^a^** | | | | | | | | | | | | | | | |
| --- | --- | --- | --- | --- | --- | --- | --- | --- | --- | --- | --- | --- | --- | --- | --- |
|  | | | | | | | | | | | | | | | |
|  | **Perceived helpfulness (being helped 'a lot')^b^** | | | | | | | | | | | | | | |
|  | **Model 1a:** Original pooled model | | | **Model 1b:** Pooled model including 3+ disorders | | | **Model 2:** lnclude the original left out category General medical-only), all included dummies subtracted by the original 7th dummy (General medical with Psychiatrist and Other mental health specialty) | | | **Model 3:** lnclude the original 7 dummies, all included dummies subtracted by the left out category (General medical-only) | | | **Final Model:** with pooled ORs from Model 2 and Model 3 | | |
|  | **OR** | **95% CI** | | **OR** | **95% CI** | | **OR** | **95% CI** | | **OR** | **95% CI** | | **OR** | **95% CI** | |
| Insurance (ref: None or unknown) | reference |  |  | reference |  |  | reference |  |  | reference |  |  | reference |  |  |
| State funded coverage or subsidized insurance | 1.33 | 0.93 | 1.92 | 1.34 | 0.93 | 1.93 | 1.33 | 0.93 | 1.92 | 1.33 | 0.93 | 1.92 | 1.33 | 0.93 | 1.92 |
| Insurance through employment or national social   security | 1.49 | 0.98 | 2.27 | 1.51 | 0.99 | 2.29 | 1.49 | 0.98 | 2.27 | 1.49 | 0.98 | 2.27 | 1.49 | 0.98 | 2.27 |
| Direct private/optional insurance | 0.85 | 0.44 | 1.67 | 0.86 | 0.44 | 1.67 | 0.85 | 0.44 | 1.67 | 0.85 | 0.44 | 1.67 | 0.85 | 0.44 | 1.67 |
| Other | 1.32 | 0.90 | 1.93 | 1.33 | 0.90 | 1.94 | 1.32 | 0.90 | 1.93 | 1.32 | 0.90 | 1.93 | 1.32 | 0.90 | 1.93 |
| *X*^2^_4_ (p-value) | 6.38 (0.172) | | | 6.54 (0.163) | | | 6.38 (0.172) | | | 6.38 (0.172) | | | 6.38 (0.172) | | |
| 12-month DSM-IV disorders |  |  |  |  |  |  |  |  |  |  |  |  |  |  |  |
| Major depressive disorder (ref: No) | 0.82 | 0.67 | 1.01 | 0.72 | 0.56 | 0.93 | 0.82 | 0.67 | 1.01 | 0.82 | 0.67 | 1.01 | 0.82 | 0.67 | 1.01 |
| *X*^2^_1_ (p-value) | 3.66 (0.056) | | | 6.33* (0.012) | | | 3.66 (0.056) | | | 3.66 (0.056) | | | 3.66 (0.056) | | |
| Bipolar disorder (ref: No) | 0.73 | 0.52 | 1.02 | 0.65 | 0.45 | 0.92 | 0.73 | 0.52 | 1.02 | 0.73 | 0.52 | 1.02 | 0.73 | 0.52 | 1.02 |
| *X*^2^_1_ (p-value) | 3.41 (0.065) | | | 5.98* (0.015) | | | 3.41 (0.065) | | | 3.41 (0.065) | | | 3.41 (0.065) | | |
| Generalized anxiety disorder (ref: No) | 0.82 | 0.63 | 1.08 | 0.73 | 0.53 | 0.99 | 0.82 | 0.63 | 1.08 | 0.82 | 0.63 | 1.08 | 0.82 | 0.63 | 1.08 |
| *X*^2^_1_ (p-value) | 2.00 (0.157) | | | 4.23* (0.040) | | | 2.00 (0.157) | | | 2.00 (0.157) | | | 2.00 (0.157) | | |
| Panic disorder/Agoraphobia (ref: No) | 0.90 | 0.72 | 1.12 | 0.79 | 0.61 | 1.02 | 0.90 | 0.72 | 1.12 | 0.90 | 0.72 | 1.12 | 0.90 | 0.72 | 1.12 |
| *X*^2^_1_ (p-value) | 0.94 (0.332) | | | 3.31 (0.069) | | | 0.94 (0.332) | | | 0.94 (0.332) | | | 0.94 (0.332) | | |
| Posttraumatic stress disorder (ref: No) | 0.98 | 0.77 | 1.24 | 0.85 | 0.65 | 1.12 | 0.98 | 0.77 | 1.24 | 0.98 | 0.77 | 1.24 | 0.98 | 0.77 | 1.24 |
| *X*^2^_1_ (p-value) | 0.04 (0.845) | | | 1.38 (0.241) | | | 0.04 (0.845) | | | 0.04 (0.845) | | | 0.04 (0.845) | | |
| Specific phobia (ref: No) | 1.21 | 0.98 | 1.49 | 1.06 | 0.82 | 1.38 | 1.21 | 0.98 | 1.49 | 1.21 | 0.98 | 1.49 | 1.21 | 0.98 | 1.49 |
| *X*^2^_1_ (p-value) | 3.07 (0.080) | | | 0.18 (0.669) | | | 3.07 (0.080) | | | 3.07 (0.080) | | | 3.07 (0.080) | | |
| Social phobia (ref: No) | 0.84 | 0.69 | 1.02 | 0.73 | 0.57 | 0.95 | 0.84 | 0.69 | 1.02 | 0.84 | 0.69 | 1.02 | 0.84 | 0.69 | 1.02 |
| *X*^2^_1_ (p-value) | 3.10 (0.078) | | | 5.68* (0.017) | | | 3.10 (0.078) | | | 3.10 (0.078) | | | 3.10 (0.078) | | |
| Substance use disorder (ref: No) | 0.97 | 0.71 | 1.31 | 0.85 | 0.61 | 1.18 | 0.97 | 0.71 | 1.31 | 0.97 | 0.71 | 1.31 | 0.97 | 0.71 | 1.31 |
| *X*^2^_1_ (p-value) | 0.05 (0.823) | | | 1.00 (0.317) | | | 0.05 (0.823) | | | 0.05 (0.823) | | | 0.05 (0.823) | | |
| Number of 12-month disorders (ref: Exactly 1 or 2) |  |  |  | reference |  |  |  |  |  |  |  |  |  |  |  |
| 3 or more |  |  |  | 1.48 | 0.98 | 2.23 |  |  |  |  |  |  |  |  |  |
| *X*^2^_1_ (p-value) |  | | | 3.49 (0.062) | | |  | | |  | | |  | | |
| Treatment profiles |  |  |  |  |  |  |  |  |  |  |  |  |  |  |  |
| General medical-only |  |  |  |  |  |  | 0.46 | 0.38 | 0.54 |  |  |  | 0.46 | 0.38 | 0.54 |
| *X*^2^_1_ (p-value) |  | | |  | | | 75.98* (<0.001) | | |  | | | 75.98* (<0.001) | | |

*Continued over.*

| **Table A4 continued. Development of logistic regression models showing joint associations of sociodemographics, disorder types, and treatment profiles with perceived helpfulness (being helped 'a lot'), among respondents with 12-month DSM-IV disorders who reported 12-month use of providers for mental health, all countries combined (N=3119)^a^** | | | | | | | | | | | | | | | | |
| --- | --- | --- | --- | --- | --- | --- | --- | --- | --- | --- | --- | --- | --- | --- | --- | --- |
|  | | | | | | | | | | | | | | | | |
|  | | **Perceived helpfulness (being helped 'a lot')^b^** | | | | | | | | | | | | | | |
|  | | **Model 1a:** Original pooled model | | | **Model 1b:** Pooled model including 3+ disorders | | | **Model 2:** lnclude the original left out category General medical-only), all included dummies subtracted by the original 7th dummy (General medical with Psychiatrist and Other mental health specialty) | | | **Model 3:** lnclude the original 7 dummies, all included dummies subtracted by the left out category (General medical-only) | | | **Final Model:** with pooled ORs from Model 2 and Model 3 | | |
|  |  | **OR** | **95% CI** | | **OR** | **95% CI** | | **OR** | **95% CI** | | **OR** | **95% CI** | | **OR** | **95% CI** | |
| Psychiatrist-only | | 1.53 | 1.13 | 2.07 | 1.54 | 1.14 | 2.09 | 0.70 | 0.54 | 0.91 | 0.70 | 0.54 | 0.91 | 0.70 | 0.54 | 0.91 |
| *X*^2^_1_ (p-value) | | 7.70* (0.006) | | | 7.81* (0.005) | | | 7.28* (0.007) | | | 7.28* (0.007) | | | 7.28* (0.007) | | |
| Other mental health specialty-only | | 1.56 | 1.20 | 2.04 | 1.57 | 1.20 | 2.04 | 0.71 | 0.58 | 0.87 | 0.71 | 0.58 | 0.87 | 0.71 | 0.58 | 0.87 |
| *X*^2^_1_ (p-value) | | 10.83* (0.001) | | | 10.88* (0.001) | | | 10.75* (0.001) | | | 10.75* (0.001) | | | 10.75* (0.001) | | |
| Spiritual/healer-only | | 2.59 | 1.78 | 3.77 | 2.60 | 1.78 | 3.78 | 1.18 | 0.87 | 1.59 | 1.18 | 0.87 | 1.59 | 1.18 | 0.87 | 1.59 |
| *X*^2^_1_ (p-value) | | 24.75* (<0.001) | | | 25.00* (<0.001) | | | 1.16 (0.282) | | | 1.16 (0.282) | | | 1.16 (0.282) | | |
| General medical with Psychiatrist | | 2.48 | 1.65 | 3.74 | 2.52 | 1.68 | 3.76 | 1.13 | 0.80 | 1.61 | 1.13 | 0.80 | 1.61 | 1.13 | 0.80 | 1.61 |
| *X*^2^_1_ (p-value) | | 19.21* (<0.001) | | | 20.29* (<0.001) | | | 0.47 (0.493) | | | 0.47 (0.493) | | | 0.47 (0.493) | | |
| General medical with Other mental health specialty | | 2.58 | 1.89 | 3.54 | 2.63 | 1.92 | 3.59 | 1.18 | 0.92 | 1.51 | 1.18 | 0.92 | 1.51 | 1.18 | 0.92 | 1.51 |
| *X*^2^_1_ (p-value) | | 35.24* (<0.001) | | | 36.77* (<0.001) | | | 1.63 (0.201) | | | 1.63 (0.201) | | | 1.63 (0.201) | | |
| Psychiatrist with Other mental health specialty | | 3.29 | 2.13 | 5.07 | 3.28 | 2.13 | 5.06 | 1.50 | 1.05 | 2.14 | 1.50 | 1.05 | 2.14 | 1.50 | 1.05 | 2.14 |
| *X*^2^_1_ (p-value) | | 29.19* (<0.001) | | | 29.05* (<0.001) | | | 4.98* (0.026) | | | 4.98* (0.026) | | | 4.98* (0.026) | | |
| General medical with Psychiatrist and Other mental  health specialty | | 4.15 | 2.83 | 6.07 | 4.15 | 2.83 | 6.08 |  |  |  | 1.89 | 1.37 | 2.61 | 1.89 | 1.37 | 2.61 |
| *X*^2^_1_ (p-value) | | 53.89* (<0.001) | | | 53.57* (<0.001) | | |  | | | 14.87* (<0.001) | | | 14.87* (<0.001) | | |
| **Pooled *X*^2^ tests** | |  | | | | | | | | | | | | | | |
| Mental disorders, *X*^2^_8_ (p-value) | | 18.53* (0.018) | | | | | | | | | | | | | | |
| Treatment profiles, *X*^2^_7_ (p-value) | | 97.76* (<0.001) | | | | | | | | | | | | | | |
| Disorders and profiles, X^2^_15_ (p-value) | | 102.27* (<0.001) | | | | | | | | | | | | | | |
| * Significant at .05 level, two-sided test. | | | | | | | | | | | | | | | | |
| All models included survey dummy variables. In the final adjusted model, the ORs associated with treatment profiles were centered to have a product of 0, allowing direct interpretation of each individual OR with the average in the total sample. The Akaike information criterion (AIC) was used to compare the fit of the final adjusted model with and without interaction terms. The additive model including controls, 8 disorder groups, and 7 treatment profile groups without interactions was the better fit for the final model (overall model AIC=2454.96; 15df for disorders and profiles *X*^2^=92.95, p<0.0001, AIC=2483.79) compared to the interaction model including controls, 8 disorder groups, and 7 treatment profile groups and 56 disorder*profile interactions (overall model AIC=2500.00; 56df for disorder*profile interactions *X*^2^=181.95, p<0.0001, AIC=2543.11). | | | | | | | | | | | | | | | | |
| *^a^* The General medical with Spiritual/healer treatment profile (n=102) was dropped in the final model since it comprised a relatively small number of patients and made the modelling unstable, hence the sample size for the model is 3119. | | | | | | | | | | | | | | | | |
| *^b^* Patient report of being helped 'a lot' by any type of provider seen. | | | | | | | | | | | | | | | | |
| *^c^* High income was defined as greater than two times the within-country median per capita family income (i.e., income divided by number of family members), high-average income as 100-200% times the median, low-average as 50–100% of the median, and low income as less than 50% of the median. | | | | | | | | | | | | | | | | |
| *^d^* In high-income countries, the high education category corresponded to a college degree, high-average to some post-secondary education without a college degree, low-average to secondary school graduation, and low to less than secondary education. These four categories comprised roughly equal sized groups. Thresholds in other countries were applied to achieve the same split. | | | | | | | | | | | | | | | | |
| **Table A5. Development of logistic regression models showing joint associations of sociodemographics, disorder types, and treatment profiles with perceived helpfulness (being helped 'a lot'), among respondents with 12-month DSM-IV disorders who reported 12-month use of providers for mental health, high-income countries (N=2546)^a^** | | | | | | | | | | | | | | | | |
|  | | | | | | | | | | | | | | | | |
|  | **Perceived helpfulness (being helped 'a lot')^b^** | | | | | | | | | | | | | | | |
|  | **Model 1a:** Original pooled model | | | | **Model 1b:** Pooled model including 3+ disorders | | | **Model 2:** lnclude the original left out category General medical-only), all included dummies subtracted by the original 7th dummy (General medical with Psychiatrist and Other mental health specialty) | | | **Model 3:** lnclude the original 7 dummies, all included dummies subtracted by the left out category (General medical-only) | | | **Final Model:** with pooled ORs from Model 2 and Model 3 | | |
|  | **OR** | | **95% CI** | | **OR** | **95% CI** | | **OR** | **95% CI** | | **OR** | **95% CI** | | **OR** | **95% CI** | |
| Gender (ref: Female) | reference | |  |  | reference |  |  | reference |  |  | reference |  |  | reference |  |  |
| Male | 0.73 | | 0.57 | 0.93 | 0.73 | 0.57 | 0.94 | 0.73 | 0.57 | 0.94 | 0.73 | 0.57 | 0.94 | 0.73 | 0.57 | 0.94 |
| *X*^2^_1_ (p-value) | 6.27* (0.012) | | | | 6.24* (0.013) | | | 6.24* (0.013) | | | 6.24* (0.013) | | | 6.24* (0.013) | | |
| Age at interview (years) (ref: ≥65) | reference | |  |  | reference |  |  | reference |  |  | reference |  |  | reference |  |  |
| ≤34 years | 0.52 | | 0.29 | 0.94 | 0.52 | 0.29 | 0.94 | 0.52 | 0.29 | 0.94 | 0.52 | 0.29 | 0.94 | 0.52 | 0.29 | 0.94 |
| 35-49 | 0.59 | | 0.35 | 1.00 | 0.60 | 0.36 | 1.01 | 0.60 | 0.36 | 1.01 | 0.60 | 0.36 | 1.01 | 0.60 | 0.36 | 1.01 |
| 50-64 | 0.96 | | 0.59 | 1.59 | 0.97 | 0.59 | 1.60 | 0.97 | 0.59 | 1.60 | 0.97 | 0.59 | 1.60 | 0.97 | 0.59 | 1.60 |
| *X*^2^_3_ (p-value) | 16.13* (0.001) | | | | 16.34* (0.001) | | | 16.34* (0.001) | | | 16.34* (0.001) | | | 16.34* (0.001) | | |
| Marital status (ref: Married/cohabitating) | reference | |  |  | reference |  |  | reference |  |  | reference |  |  | reference |  |  |
| Separated/widowed/divorced | 0.95 | | 0.74 | 1.21 | 0.96 | 0.75 | 1.22 | 0.96 | 0.75 | 1.22 | 0.96 | 0.75 | 1.22 | 0.96 | 0.75 | 1.22 |
| Never married | 1.10 | | 0.84 | 1.44 | 1.10 | 0.84 | 1.45 | 1.10 | 0.84 | 1.45 | 1.10 | 0.84 | 1.45 | 1.10 | 0.84 | 1.45 |
| *X*^2^_2_ (p-value) | 0.78 (0.677) | | | | 0.73 (0.693) | | | 0.73 (0.693) | | | 0.73 (0.693) | | | 0.73 (0.693) | | |
| Family income^c^ (ref: High) | reference | |  |  | reference |  |  | reference |  |  | reference |  |  | reference |  |  |
| Low | 0.84 | | 0.63 | 1.13 | 0.85 | 0.63 | 1.14 | 0.85 | 0.63 | 1.14 | 0.85 | 0.63 | 1.14 | 0.85 | 0.63 | 1.14 |
| Low-average | 0.93 | | 0.71 | 1.22 | 0.94 | 0.71 | 1.23 | 0.94 | 0.71 | 1.23 | 0.94 | 0.71 | 1.23 | 0.94 | 0.71 | 1.23 |
| High-average | 0.86 | | 0.65 | 1.15 | 0.86 | 0.65 | 1.15 | 0.86 | 0.65 | 1.15 | 0.86 | 0.65 | 1.15 | 0.86 | 0.65 | 1.15 |
| *X*^2^_3_ (p-value) | 1.65 (0.648) | | | | 1.55 (0.671) | | | 1.55 (0.671) | | | 1.55 (0.671) | | | 1.55 (0.671) | | |
| Education^d^ (ref: High) | reference | |  |  | reference |  |  | reference |  |  | reference |  |  | reference |  |  |
| Low | 1.05 | | 0.75 | 1.46 | 1.05 | 0.75 | 1.46 | 1.05 | 0.75 | 1.46 | 1.05 | 0.75 | 1.46 | 1.05 | 0.75 | 1.46 |
| Low-average | 0.93 | | 0.69 | 1.26 | 0.92 | 0.68 | 1.25 | 0.92 | 0.68 | 1.25 | 0.92 | 0.68 | 1.25 | 0.92 | 0.68 | 1.25 |
| High-average | 0.86 | | 0.67 | 1.10 | 0.86 | 0.67 | 1.10 | 0.86 | 0.67 | 1.10 | 0.86 | 0.67 | 1.10 | 0.86 | 0.67 | 1.10 |
| *X*^2^_3_ (p-value) | 2.20 (0.532) | | | | 2.13 (0.546) | | | 2.13 (0.546) | | | 2.13 (0.546) | | | 2.13 (0.546) | | |
| Employment (ref: Working) | reference | |  |  | reference |  |  | reference |  |  | reference |  |  | reference |  |  |
| Homemaker | 0.92 | | 0.64 | 1.32 | 0.93 | 0.65 | 1.34 | 0.93 | 0.65 | 1.34 | 0.93 | 0.65 | 1.34 | 0.93 | 0.65 | 1.34 |
| Retired | 0.97 | | 0.61 | 1.55 | 0.98 | 0.62 | 1.56 | 0.98 | 0.62 | 1.56 | 0.98 | 0.62 | 1.56 | 0.98 | 0.62 | 1.56 |
| Student | 0.79 | | 0.48 | 1.31 | 0.79 | 0.48 | 1.30 | 0.79 | 0.48 | 1.30 | 0.79 | 0.48 | 1.30 | 0.79 | 0.48 | 1.30 |
| Other | 0.81 | | 0.60 | 1.09 | 0.81 | 0.60 | 1.09 | 0.81 | 0.60 | 1.09 | 0.81 | 0.60 | 1.09 | 0.81 | 0.60 | 1.09 |
| *X*^2^_4_ (p-value) | 2.62 (0.624) | | | | 2.60 (0.627) | | | 2.60 (0.627) | | | 2.60 (0.627) | | | 2.60 (0.627) | | |

*Continued over.*

| **Table A5 continued. Development of logistic regression models showing joint associations of sociodemographics, disorder types, and treatment profiles with perceived helpfulness (being helped 'a lot'), among respondents with 12-month DSM-IV disorders who reported 12-month use of providers for mental health, high-income countries (N=2546)^a^** | | | | | | | | | | | | | | | |
| --- | --- | --- | --- | --- | --- | --- | --- | --- | --- | --- | --- | --- | --- | --- | --- |
|  | | | | | | | | | | | | | | | |
|  | **Perceived helpfulness (being helped 'a lot')^b^** | | | | | | | | | | | | | | |
|  | **Model 1a:** Original pooled model | | | **Model 1b:** Pooled model including 3+ disorders | | | **Model 2:** lnclude the original left out category General medical-only), all included dummies subtracted by the original 7th dummy (General medical with Psychiatrist and Other mental health specialty) | | | **Model 3:** lnclude the original 7 dummies, all included dummies subtracted by the left out category (General medical-only) | | | **Final Model:** with pooled ORs from Model 2 and Model 3 | | |
|  | **OR** | **95% CI** | | **OR** | **95% CI** | | **OR** | **95% CI** | | **OR** | **95% CI** | | **OR** | **95% CI** | |
| Insurance (ref: None or unknown) | reference |  |  | reference |  |  | reference |  |  | reference |  |  | reference |  |  |
| State funded coverage or subsidized insurance | 1.19 | 0.79 | 1.79 | 1.20 | 0.80 | 1.79 | 1.20 | 0.80 | 1.79 | 1.20 | 0.80 | 1.79 | 1.20 | 0.80 | 1.79 |
| Insurance through employment or national social   security | 1.51 | 0.96 | 2.38 | 1.52 | 0.96 | 2.40 | 1.52 | 0.96 | 2.40 | 1.52 | 0.96 | 2.40 | 1.52 | 0.96 | 2.40 |
| Direct private/optional insurance | 1.09 | 0.46 | 2.55 | 1.08 | 0.46 | 2.53 | 1.08 | 0.46 | 2.53 | 1.08 | 0.46 | 2.53 | 1.08 | 0.46 | 2.53 |
| Other | 1.31 | 0.86 | 1.98 | 1.31 | 0.87 | 1.99 | 1.31 | 0.87 | 1.99 | 1.31 | 0.87 | 1.99 | 1.31 | 0.87 | 1.99 |
| *X*^2^_4_ (p-value) | 3.96 (0.411) | | | 4.08 (0.395) | | | 4.08 (0.395) | | | 4.08 (0.395) | | | 4.08 (0.395) | | |
| 12-month DSM-IV disorders |  |  |  |  |  |  |  |  |  |  |  |  |  |  |  |
| Major depressive disorder (ref: No) | 0.87 | 0.70 | 1.09 | 0.74 | 0.56 | 0.99 | 0.74 | 0.56 | 0.99 | 0.74 | 0.56 | 0.99 | 0.74 | 0.56 | 0.99 |
| *X*^2^_1_ (p-value) | 1.45 (0.228) | | | 4.25* (0.039) | | | 4.25* (0.039) | | | 4.25* (0.039) | | | 4.25* (0.039) | | |
| Bipolar disorder (ref: No) | 0.75 | 0.52 | 1.06 | 0.64 | 0.44 | 0.93 | 0.64 | 0.44 | 0.93 | 0.64 | 0.44 | 0.93 | 0.64 | 0.44 | 0.93 |
| *X*^2^_1_ (p-value) | 2.69 (0.101) | | | 5.62* (0.018) | | | 5.62* (0.018) | | | 5.62* (0.018) | | | 5.62* (0.018) | | |
| Generalized anxiety disorder (ref: No) | 0.85 | 0.64 | 1.13 | 0.72 | 0.53 | 1.00 | 0.72 | 0.53 | 1.00 | 0.72 | 0.53 | 1.00 | 0.72 | 0.53 | 1.00 |
| *X*^2^_1_ (p-value) | 1.32 (0.251) | | | 3.98* (0.046) | | | 3.98* (0.046) | | | 3.98* (0.046) | | | 3.98* (0.046) | | |
| Panic disorder/Agoraphobia (ref: No) | 0.86 | 0.68 | 1.08 | 0.73 | 0.55 | 0.96 | 0.73 | 0.55 | 0.96 | 0.73 | 0.55 | 0.96 | 0.73 | 0.55 | 0.96 |
| *X*^2^_1_ (p-value) | 1.69 (0.194) | | | 4.99* (0.025) | | | 4.99* (0.025) | | | 4.99* (0.025) | | | 4.99* (0.025) | | |
| Posttraumatic stress disorder (ref: No) | 0.98 | 0.77 | 1.26 | 0.83 | 0.62 | 1.10 | 0.83 | 0.62 | 1.10 | 0.83 | 0.62 | 1.10 | 0.83 | 0.62 | 1.10 |
| *X*^2^_1_ (p-value) | 0.02 (0.886) | | | 1.70 (0.192) | | | 1.70 (0.192) | | | 1.70 (0.192) | | | 1.70 (0.192) | | |
| Specific phobia (ref: No) | 1.22 | 0.96 | 1.54 | 1.03 | 0.76 | 1.39 | 1.03 | 0.76 | 1.39 | 1.03 | 0.76 | 1.39 | 1.03 | 0.76 | 1.39 |
| *X*^2^_1_ (p-value) | 2.53 (0.112) | | | 0.04 (0.842) | | | 0.04 (0.842) | | | 0.04 (0.842) | | | 0.04 (0.842) | | |
| Social phobia (ref: No) | 0.81 | 0.65 | 0.99 | 0.68 | 0.52 | 0.90 | 0.68 | 0.52 | 0.90 | 0.68 | 0.52 | 0.90 | 0.68 | 0.52 | 0.90 |
| *X*^2^_1_ (p-value) | 4.20 (0.040*) | | | 7.64* (0.006) | | | 7.64* (0.006) | | | 7.64* (0.006) | | | 7.64* (0.006) | | |
| Substance use disorder (ref: No) | 1.06 | 0.76 | 1.48 | 0.90 | 0.63 | 1.28 | 0.90 | 0.63 | 1.28 | 0.90 | 0.63 | 1.28 | 0.90 | 0.63 | 1.28 |
| *X*^2^_1_ (p-value) | 0.11 (0.744) | | | 0.37 (0.542) | | | 0.37 (0.542) | | | 0.37 (0.542) | | | 0.37 (0.542) | | |
| Number of 12-month disorders (ref: Exactly 1 or 2) |  |  |  | reference |  |  | reference |  |  | reference |  |  | reference |  |  |
| 3 or more |  |  |  | 1.62 | 1.03 | 2.55 | 1.62 | 1.03 | 2.55 | 1.62 | 1.03 | 2.55 | 1.62 | 1.03 | 2.55 |
| *X*^2^_1_ (p-value) |  | | | 4.33* (0.037) | | | 4.33* (0.037) | | | 4.33* (0.037) | | | 4.33* (0.037) | | |
| Treatment profiles |  |  |  |  |  |  |  |  |  |  |  |  |  |  |  |
| General medical-only |  |  |  |  |  |  | 0.49 | 0.40 | 0.59 |  |  |  | 0.49 | 0.40 | 0.59 |
| *X*^2^_1_ (p-value) |  | | |  | | | 53.38* (<0.001) | | |  | | | 53.38* (<0.001) | | |

*Continued over.*

| **Table A5 continued. Development of logistic regression models showing joint associations of sociodemographics, disorder types, and treatment profiles with perceived helpfulness (being helped 'a lot'), among respondents with 12-month DSM-IV disorders who reported 12-month use of providers for mental health, high-income countries (N=2546)^a^** | | | | | | | | | | | | | | | |
| --- | --- | --- | --- | --- | --- | --- | --- | --- | --- | --- | --- | --- | --- | --- | --- |
|  | | | | | | | | | | | | | | | |
|  | **Perceived helpfulness (being helped 'a lot')^b^** | | | | | | | | | | | | | | |
|  | **Model 1a:** Original pooled model | | | **Model 1b:** Pooled model including 3+ disorders | | | **Model 2:** lnclude the original left out category General medical-only), all included dummies subtracted by the original 7th dummy (General medical with Psychiatrist and Other mental health specialty) | | | **Model 3:** lnclude the original 7 dummies, all included dummies subtracted by the left out category (General medical-only) | | | **Final Model:** with pooled ORs from Model 2 and Model 3 | | |
|  | **OR** | **95% CI** | | **OR** | **95% CI** | | **OR** | **95% CI** | | **OR** | **95% CI** | | **OR** | **95% CI** | |
| Psychiatrist-only | 1.26 | 0.88 | 1.79 | 1.26 | 0.88 | 1.80 | 0.62 | 0.45 | 0.85 | 0.62 | 0.45 | 0.85 | 0.62 | 0.45 | 0.85 |
| *X*^2^_1_ (p-value) | 1.61 (0.205) | | | 1.64 (0.200) | | | 8.88* (0.003) | | | 8.88* (0.003) | | | 8.88* (0.003) | | |
| Other mental health specialty-only | 1.47 | 1.10 | 1.95 | 1.47 | 1.11 | 1.96 | 0.72 | 0.58 | 0.90 | 0.72 | 0.58 | 0.90 | 0.72 | 0.58 | 0.90 |
| *X*^2^_1_ (p-value) | 6.91* (0.009) | | | 7.13* (0.008) | | | 8.59* (0.003) | | | 8.59* (0.003) | | | 8.59* (0.003) | | |
| Spiritual/healer-only | 2.51 | 1.60 | 3.91 | 2.52 | 1.61 | 3.94 | 1.23 | 0.86 | 1.76 | 1.23 | 0.86 | 1.76 | 1.23 | 0.86 | 1.76 |
| *X*^2^_1_ (p-value) | 16.42* (<0.001) | | | 16.54* (<0.001) | | | 1.29 (0.257) | | | 1.29 (0.257) | | | 1.29 (0.257) | | |
| General medical with Psychiatrist | 2.42 | 1.58 | 3.71 | 2.46 | 1.61 | 3.75 | 1.20 | 0.84 | 1.72 | 1.20 | 0.84 | 1.72 | 1.20 | 0.84 | 1.72 |
| *X*^2^_1_ (p-value) | 16.44* (<0.001) | | | 17.57* (<0.001) | | | 0.97 (0.324) | | | 0.97 (0.324) | | | 0.97 (0.324) | | |
| General medical with Other mental health specialty | 2.60 | 1.88 | 3.60 | 2.67 | 1.94 | 3.69 | 1.30 | 1.02 | 1.67 | 1.30 | 1.02 | 1.67 | 1.30 | 1.02 | 1.67 |
| *X*^2^_1_ (p-value) | 33.63* (<0.001) | | | 36.22* (<0.001) | | | 4.35* (0.037) | | | 4.35* (0.037) | | | 4.35* (0.037) | | |
| Psychiatrist with Other mental health specialty | 2.67 | 1.62 | 4.39 | 2.67 | 1.62 | 4.40 | 1.30 | 0.86 | 1.97 | 1.30 | 0.86 | 1.97 | 1.30 | 0.86 | 1.97 |
| *X*^2^_1_ (p-value) | 15.03* (<0.001) | | | 15.01* (<0.001) | | | 1.59 (0.207) | | | 1.59 (0.207) | | | 1.59 (0.207) | | |
| General medical with Psychiatrist and Other mental  health specialty | 3.80 | 2.56 | 5.63 | 3.80 | 2.56 | 5.64 |  |  |  | 1.85 | 1.32 | 2.60 | 1.85 | 1.32 | 2.60 |
| *X*^2^_1_ (p-value) | 44.49* (<0.001) | | | 44.03* (<0.001) | | |  | | | 12.79* (<0.001) | | | 12.79* (<0.001) | | |
| **Pooled *X*^2^ tests** |  | | | | | | | | | | | | | | |
| Mental disorders, *X*^2^_8_ (p-value) | 16.59* (0.035) | | | | | | | | | | | | | | |
| Treatment profiles, *X*^2^_7_ (p-value) | 77.15* (<0.001) | | | | | | | | | | | | | | |
| Disorders and profiles, X^2^_15_ (p-value) | 82.66* (<0.001) | | | | | | | | | | | | | | |
| * Significant at .05 level, two-sided test. | | | | | | | | | | | | | | | |
| All models included survey dummy variables. In the final adjusted model, the ORs associated with treatment profiles were centered to have a product of 0, allowing direct interpretation of each individual OR with the average in the total sample. | | | | | | | | | | | | | | | |
| *^a^* The General medical with Spiritual/healer treatment profile was dropped in the final model since it comprised a relatively small number of patients and made the modelling unstable, hence the sample size for the model is 2546. | | | | | | | | | | | | | | | |
| *^b^* Patient report of being helped 'a lot' by any type of provider seen. | | | | | | | | | | | | | | | |
| *^c^* High income was defined as greater than two times the within-country median per capita family income (i.e., income divided by number of family members), high-average income as 100-200% times the median, low-average as 50–100% of the median, and low income as less than 50% of the median. | | | | | | | | | | | | | | | |
| *^d^* In high-income countries, the high education category corresponded to a college degree, high-average to some post-secondary education without a college degree, low-average to secondary school graduation, and low to less than secondary education. These four categories comprised roughly equal sized groups. Thresholds in other countries were applied to achieve the same split. | | | | | | | | | | | | | | | |

| **Table A6. Development of logistic regression models showing joint associations of sociodemographics, disorder types, and treatment profiles with perceived helpfulness (being helped 'a lot'), among respondents with 12-month DSM-IV disorders who reported 12-month use of providers for mental health, low/middle-income countries (N=573)^a^** | | | | | | | | | | | | | | | |
| --- | --- | --- | --- | --- | --- | --- | --- | --- | --- | --- | --- | --- | --- | --- | --- |
|  | | | | | | | | | | | | | | | |
|  | **Perceived helpfulness (being helped 'a lot')^b^** | | | | | | | | | | | | | | |
|  | **Model 1a:** Original pooled model | | | **Model 1b:** Pooled model including 3+ disorders | | | **Model 2:** lnclude the original left out category General medical-only), all included dummies subtracted by the original 7th dummy (General medical with Psychiatrist and Other mental health specialty) | | | **Model 3:** lnclude the original 7 dummies, all included dummies subtracted by the left out category (General medical-only) | | | **Final Model:** with pooled ORs from Model 2 and Model 3 | | |
|  | **OR** | **95% CI** | | **OR** | **95% CI** | | **OR** | **95% CI** | | **OR** | **95% CI** | | **OR** | **95% CI** | |
| Gender (ref: Female) | reference |  |  | reference |  |  | reference |  |  | reference |  |  | reference |  |  |
| Male | 1.09 | 0.57 | 2.08 | 1.09 | 0.57 | 2.08 | 1.09 | 0.57 | 2.08 | 1.09 | 0.57 | 2.08 | 1.09 | 0.57 | 2.08 |
| *X*^2^_1_ (p-value) | 0.07 (0.792) | | | 0.07 (0.798) | | | 0.07 (0.792) | | | 0.07 (0.792) | | | 0.07 (0.792) | | |
| Age at interview (years) (ref: ≥65) | reference |  |  | reference |  |  | reference |  |  | reference |  |  | reference |  |  |
| ≤34 years | 1.51 | 0.51 | 4.50 | 1.46 | 0.50 | 4.31 | 1.51 | 0.51 | 4.50 | 1.51 | 0.51 | 4.50 | 1.51 | 0.51 | 4.50 |
| 35-49 | 3.30 | 1.28 | 8.55 | 3.23 | 1.27 | 8.25 | 3.30 | 1.28 | 8.55 | 3.30 | 1.28 | 8.55 | 3.30 | 1.28 | 8.55 |
| 50-64 | 3.07 | 1.18 | 8.04 | 3.00 | 1.16 | 7.78 | 3.07 | 1.18 | 8.04 | 3.07 | 1.18 | 8.04 | 3.07 | 1.18 | 8.04 |
| *X*^2^_3_ (p-value) | 12.03* (0.007) | | | 12.10* (0.007) | | | 12.03* (0.007) | | | 12.03* (0.007) | | | 12.03* (0.007) | | |
| Marital status (ref: Married/cohabitating) | reference |  |  | reference |  |  | reference |  |  | reference |  |  | reference |  |  |
| Separated/widowed/divorced | 0.89 | 0.52 | 1.52 | 0.89 | 0.52 | 1.51 | 0.89 | 0.52 | 1.52 | 0.89 | 0.52 | 1.52 | 0.89 | 0.52 | 1.52 |
| Never married | 0.81 | 0.42 | 1.57 | 0.81 | 0.41 | 1.58 | 0.81 | 0.42 | 1.57 | 0.81 | 0.42 | 1.57 | 0.81 | 0.42 | 1.57 |
| *X*^2^_2_ (p-value) | 0.48 (0.785) | | | 0.47 (0.789) | | | 0.48 (0.785) | | | 0.48 (0.785) | | | 0.48 (0.785) | | |
| Family income^c^ (ref: High) | reference |  |  | reference |  |  | reference |  |  | reference |  |  | reference |  |  |
| Low | 1.02 | 0.50 | 2.10 | 1.03 | 0.50 | 2.12 | 1.02 | 0.50 | 2.10 | 1.02 | 0.50 | 2.10 | 1.02 | 0.50 | 2.10 |
| Low-average | 0.63 | 0.35 | 1.14 | 0.65 | 0.36 | 1.18 | 0.63 | 0.35 | 1.14 | 0.63 | 0.35 | 1.14 | 0.63 | 0.35 | 1.14 |
| High-average | 1.06 | 0.52 | 2.14 | 1.06 | 0.52 | 2.15 | 1.06 | 0.52 | 2.14 | 1.06 | 0.52 | 2.14 | 1.06 | 0.52 | 2.14 |
| *X*^2^_3_ (p-value) | 3.40 (0.334) | | | 3.11 (0.375) | | | 3.40 (0.334) | | | 3.40 (0.334) | | | 3.40 (0.334) | | |
| Education^d^ (ref: High) | reference |  |  | reference |  |  | reference |  |  | reference |  |  | reference |  |  |
| Low | 1.02 | 0.52 | 2.02 | 1.00 | 0.50 | 2.00 | 1.02 | 0.52 | 2.02 | 1.02 | 0.52 | 2.02 | 1.02 | 0.52 | 2.02 |
| Low-average | 1.07 | 0.54 | 2.13 | 1.08 | 0.54 | 2.13 | 1.07 | 0.54 | 2.13 | 1.07 | 0.54 | 2.13 | 1.07 | 0.54 | 2.13 |
| High-average | 1.04 | 0.55 | 1.97 | 1.04 | 0.55 | 1.96 | 1.04 | 0.55 | 1.97 | 1.04 | 0.55 | 1.97 | 1.04 | 0.55 | 1.97 |
| *X*^2^_3_ (p-value) | 0.04 (0.998) | | | 0.08 (0.995) | | | 0.04 (0.998) | | | 0.04 (0.998) | | | 0.04 (0.998) | | |
| Employment (ref: Working) | reference |  |  | reference |  |  | reference |  |  | reference |  |  | reference |  |  |
| Homemaker | 0.72 | 0.35 | 1.49 | 0.72 | 0.35 | 1.48 | 0.72 | 0.35 | 1.49 | 0.72 | 0.35 | 1.49 | 0.72 | 0.35 | 1.49 |
| Retired | 0.90 | 0.30 | 2.67 | 0.92 | 0.31 | 2.73 | 0.90 | 0.30 | 2.67 | 0.90 | 0.30 | 2.67 | 0.90 | 0.30 | 2.67 |
| Student | 0.76 | 0.25 | 2.33 | 0.75 | 0.25 | 2.31 | 0.76 | 0.25 | 2.33 | 0.76 | 0.25 | 2.33 | 0.76 | 0.25 | 2.33 |
| Other | 0.48 | 0.25 | 0.93 | 0.48 | 0.25 | 0.94 | 0.48 | 0.25 | 0.93 | 0.48 | 0.25 | 0.93 | 0.48 | 0.25 | 0.93 |
| *X*^2^_4_ (p-value) | 5.23 (0.264) | | | 5.20 (0.268) | | | 5.23 (0.264) | | | 5.23 (0.264) | | | 5.23 (0.264) | | |

*Continued over.*

| **Table A6 continued. Development of logistic regression models showing joint associations of sociodemographics, disorder types, and treatment profiles with perceived helpfulness (being helped 'a lot'), among respondents with 12-month DSM-IV disorders who reported 12-month use of providers for mental health, low/middle-income countries (N=573)^a^** | | | | | | | | | | | | | | | |
| --- | --- | --- | --- | --- | --- | --- | --- | --- | --- | --- | --- | --- | --- | --- | --- |
|  | | | | | | | | | | | | | | | |
|  | **Perceived helpfulness (being helped 'a lot')^b^** | | | | | | | | | | | | | | |
|  | **Model 1a:** Original pooled model | | | **Model 1b:** Pooled model including 3+ disorders | | | **Model 2:** lnclude the original left out category General medical-only), all included dummies subtracted by the original 7th dummy (General medical with Psychiatrist and Other mental health specialty) | | | **Model 3:** lnclude the original 7 dummies, all included dummies subtracted by the left out category (General medical-only) | | | **Final Model:** with pooled ORs from Model 2 and Model 3 | | |
|  | **OR** | **95% CI** | | **OR** | **95% CI** | | **OR** | **95% CI** | | **OR** | **95% CI** | | **OR** | **95% CI** | |
| Insurance (ref: None or unknown) | reference |  |  | reference |  |  | reference |  |  | reference |  |  | reference |  |  |
| State funded coverage or subsidized insurance | 2.62 | 1.07 | 6.42 | 2.59 | 1.06 | 6.35 | 2.62 | 1.07 | 6.42 | 2.62 | 1.07 | 6.42 | 2.62 | 1.07 | 6.42 |
| Insurance through employment or national social   security | 2.46 | 0.77 | 7.86 | 2.45 | 0.77 | 7.79 | 2.46 | 0.77 | 7.86 | 2.46 | 0.77 | 7.86 | 2.46 | 0.77 | 7.86 |
| Direct private/optional insurance | 0.64 | 0.22 | 1.87 | 0.62 | 0.21 | 1.81 | 0.64 | 0.22 | 1.87 | 0.64 | 0.22 | 1.87 | 0.64 | 0.22 | 1.87 |
| Other | 1.52 | 0.59 | 3.91 | 1.50 | 0.58 | 3.87 | 1.52 | 0.59 | 3.91 | 1.52 | 0.59 | 3.91 | 1.52 | 0.59 | 3.91 |
| *X*^2^_4_ (p-value) | 10.84* (0.028) | | | 10.99* (0.027) | | | 10.84* (0.028) | | | 10.84* (0.028) | | | 10.84* (0.028) | | |
| 12-month DSM-IV disorders |  |  |  |  |  |  |  |  |  |  |  |  |  |  |  |
| Major depressive disorder (ref: No) | 0.54 | 0.31 | 0.92 | 0.59 | 0.31 | 1.10 | 0.54 | 0.31 | 0.92 | 0.54 | 0.31 | 0.92 | 0.54 | 0.31 | 0.92 |
| *X*^2^_1_ (p-value) | 5.22* (0.022) | | | 2.84 (0.092) | | | 5.22* (0.022) | | | 5.22* (0.022) | | | 5.22* (0.022) | | |
| Bipolar disorder (ref: No) | 0.59 | 0.21 | 1.65 | 0.66 | 0.22 | 1.96 | 0.59 | 0.21 | 1.65 | 0.59 | 0.21 | 1.65 | 0.59 | 0.21 | 1.65 |
| *X*^2^_1_ (p-value) | 1.02 (0.313) | | | 0.57 (0.450) | | | 1.02 (0.313) | | | 1.02 (0.313) | | | 1.02 (0.313) | | |
| Generalized anxiety disorder (ref: No) | 0.60 | 0.24 | 1.51 | 0.66 | 0.23 | 1.89 | 0.60 | 0.24 | 1.51 | 0.60 | 0.24 | 1.51 | 0.60 | 0.24 | 1.51 |
| *X*^2^_1_ (p-value) | 1.19 (0.276) | | | 0.61 (0.434) | | | 1.19 (0.276) | | | 1.19 (0.276) | | | 1.19 (0.276) | | |
| Panic disorder/Agoraphobia (ref: No) | 0.94 | 0.52 | 1.69 | 1.06 | 0.51 | 2.19 | 0.94 | 0.52 | 1.69 | 0.94 | 0.52 | 1.69 | 0.94 | 0.52 | 1.69 |
| *X*^2^_1_ (p-value) | 0.05 (0.823) | | | 0.02 (0.879) | | | 0.05 (0.823) | | | 0.05 (0.823) | | | 0.05 (0.823) | | |
| Posttraumatic stress disorder (ref: No) | 1.00 | 0.48 | 2.11 | 1.11 | 0.48 | 2.53 | 1.00 | 0.48 | 2.11 | 1.00 | 0.48 | 2.11 | 1.00 | 0.48 | 2.11 |
| *X*^2^_1_ (p-value) | <0.001 (0.995) | | | 0.06 (0.811) | | | <0.001 (0.995) | | | <0.001 (0.995) | | | <0.001 (0.995) | | |
| Specific phobia (ref: No) | 0.99 | 0.63 | 1.56 | 1.08 | 0.61 | 1.92 | 0.99 | 0.63 | 1.56 | 0.99 | 0.63 | 1.56 | 0.99 | 0.63 | 1.56 |
| *X*^2^_1_ (p-value) | 0.002 (0.969) | | | 0.08 (0.782) | | | 0.002 (0.969) | | | 0.002 (0.969) | | | 0.002 (0.969) | | |
| Social phobia (ref: No) | 1.22 | 0.67 | 2.23 | 1.40 | 0.67 | 2.90 | 1.22 | 0.67 | 2.23 | 1.22 | 0.67 | 2.23 | 1.22 | 0.67 | 2.23 |
| *X*^2^_1_ (p-value) | 0.45 (0.502) | | | 0.82 (0.366) | | | 0.45 (0.502) | | | 0.45 (0.502) | | | 0.45 (0.502) | | |
| Substance use disorder (ref: No) | 0.53 | 0.22 | 1.24 | 0.58 | 0.23 | 1.49 | 0.53 | 0.22 | 1.24 | 0.53 | 0.22 | 1.24 | 0.53 | 0.22 | 1.24 |
| *X*^2^_1_ (p-value) | 2.22 (0.137) | | | 1.30 (0.254) | | | 2.22 (0.137) | | | 2.22 (0.137) | | | 2.22 (0.137) | | |
| Number of 12-month disorders (ref: Exactly 1 or 2) |  |  |  | reference |  |  |  |  |  |  |  |  |  |  |  |
| 3 or more |  |  |  | 0.72 | 0.27 | 1.91 |  |  |  |  |  |  |  |  |  |
| *X*^2^_1_ (p-value) |  | | | 0.44 (0.507) | | |  | | |  | | |  | | |
| Treatment profiles |  |  |  |  |  |  |  |  |  |  |  |  |  |  |  |
| General medical-only |  |  |  |  |  |  | 0.28 | 0.17 | 0.44 |  |  |  | 0.28 | 0.17 | 0.44 |
| *X*^2^_1_ (p-value) |  |  |  |  |  |  | 29.71* (<0.001) | | |  | | | 29.71* (<0.001) | | |

*Continued over.*

| **Table A6 continued. Development of logistic regression models showing joint associations of sociodemographics, disorder types, and treatment profiles with perceived helpfulness (being helped 'a lot'), among respondents with 12-month DSM-IV disorders who reported 12-month use of providers for mental health, low/middle-income countries (N=573)^a^** | | | | | | | | | | | | | | | |
| --- | --- | --- | --- | --- | --- | --- | --- | --- | --- | --- | --- | --- | --- | --- | --- |
|  | | | | | | | | | | | | | | | |
|  | **Perceived helpfulness (being helped 'a lot')^b^** | | | | | | | | | | | | | | |
|  | **Model 1a:** Original pooled model | | | **Model 1b:** Pooled model including 3+ disorders | | | **Model 2:** lnclude the original left out category General medical-only), all included dummies subtracted by the original 7th dummy (General medical with Psychiatrist and Other mental health specialty) | | | **Model 3:** lnclude the original 7 dummies, all included dummies subtracted by the left out category (General medical-only) | | | **Final Model:** with pooled ORs from Model 2 and Model 3 | | |
|  | **OR** | **95% CI** | | **OR** | **95% CI** | | **OR** | **95% CI** | | **OR** | **95% CI** | | **OR** | **95% CI** | |
| Psychiatrist-only | 2.72 | 1.43 | 5.21 | 2.73 | 1.43 | 5.23 | 0.76 | 0.42 | 1.35 | 0.76 | 0.42 | 1.35 | 0.76 | 0.42 | 1.35 |
| *X*^2^_1_ (p-value) | 9.38* (0.002) | | | 9.35* (0.002) | | | 0.92 (0.338) | | | 0.92 (0.338) | | | 0.92 (0.338) | | |
| Other mental health specialty-only | 2.52 | 1.29 | 4.92 | 2.54 | 1.31 | 4.95 | 0.70 | 0.42 | 1.17 | 0.70 | 0.42 | 1.17 | 0.70 | 0.42 | 1.17 |
| *X*^2^_1_ (p-value) | 7.47* (0.006) | | | 7.69* (0.006) | | | 1.92 (0.166) | | | 1.92 (0.166) | | | 1.92 (0.166) | | |
| Spiritual/healer-only | 3.66 | 1.85 | 7.25 | 3.66 | 1.83 | 7.32 | 1.02 | 0.53 | 1.94 | 1.02 | 0.53 | 1.94 | 1.02 | 0.53 | 1.94 |
| *X*^2^_1_ (p-value) | 14.11* (<0.001) | | | 13.72* (<0.001) | | | 0.002 (0.963) | | | 0.002 (0.963) | | | 0.002 (0.963) | | |
| General medical with Psychiatrist | 3.32 | 0.92 | 12.02 | 3.33 | 0.92 | 12.08 | 0.92 | 0.27 | 3.11 | 0.92 | 0.27 | 3.11 | 0.92 | 0.27 | 3.11 |
| *X*^2^_1_ (p-value) | 3.42 (0.064) | | | 3.42 (0.064) | | | 0.02 (0.895) | | | 0.02 (0.895) | | | 0.02 (0.895) | | |
| General medical with Other mental health specialty | 2.57 | 0.66 | 9.95 | 2.67 | 0.69 | 10.38 | 0.71 | 0.23 | 2.25 | 0.71 | 0.23 | 2.25 | 0.71 | 0.23 | 2.25 |
| *X*^2^_1_ (p-value) | 1.91 (0.9998) | | | 2.04 (0.153) | | | 0.34 (0.560) | | | 0.34 (0.560) | | | 0.34 (0.560) | | |
| Psychiatrist with Other mental health specialty | 9.10 | 3.98 | 20.78 | 9.21 | 4.04 | 20.96 | 2.52 | 1.29 | 4.94 | 2.52 | 1.29 | 4.94 | 2.52 | 1.29 | 4.94 |
| *X*^2^_1_ (p-value) | 28.01* (<0.001) | | | 28.52* (<0.001) | | | 7.45* (0.006) | | | 7.45* (0.006) | | | 7.45* (0.006) | | |
| General medical with Psychiatrist and Other mental  health specialty | 14.60 | 3.23 | 66.02 | 14.22 | 3.15 | 64.17 |  |  |  | 4.05 | 1.16 | 14.17 | 4.05 | 1.16 | 14.17 |
| *X*^2^_1_ (p-value) | 12.37* (<0.001) | | | 12.17* (<0.001) | | |  | | | 4.88* (0.027) | | | 4.88* (0.027) | | |
| **Pooled *X*^2^ tests** |  | | | | | | | | | | | | | | |
| Mental disorders, *X*^2^_8_ (p-value) | 11.33 (0.180) | | | | | | | | | | | | | | |
| Treatment profiles, *X*^2^_7_ (p-value) | 42.13* (<0.001) | | | | | | | | | | | | | | |
| Disorders and profiles, X^2^_15_ (p-value) | 50.27* (<0.001) | | | | | | | | | | | | | | |
| * Significant at .05 level, two-sided test. | | | | | | | | | | | | | | | |
| All models included survey dummy variables. In the final adjusted model, the ORs associated with treatment profiles were centered to have a product of 0, allowing direct interpretation of each individual OR with the average in the total sample. | | | | | | | | | | | | | | | |
| *^a^* The General medical with Spiritual/healer treatment profile was dropped in the final model since it comprised a relatively small number of patients and made the modelling unstable, hence the sample size for the model is 573. | | | | | | | | | | | | | | | |
| *^b^* Patient report of being helped 'a lot' by any type of provider seen. | | | | | | | | | | | | | | | |
| *^c^* High income was defined as greater than two times the within-country median per capita family income (i.e., income divided by number of family members), high-average income as 100-200% times the median, low-average as 50–100% of the median, and low income as less than 50% of the median. | | | | | | | | | | | | | | | |
| *^d^* In high-income countries, the high education category corresponded to a college degree, high-average to some post-secondary education without a college degree, low-average to secondary school graduation, and low to less than secondary education. These four categories comprised roughly equal sized groups. Thresholds in other countries were applied to achieve the same split. | | | | | | | | | | | | | | | |
